# Isotocin Regulates Growth Hormone but Not Prolactin Release From the Pituitary of Ricefield Eels

**DOI:** 10.3389/fendo.2018.00166

**Published:** 2018-04-12

**Authors:** Wei Yang, Ning Zhang, Boyang Shi, Shen Zhang, Lihong Zhang, Weimin Zhang

**Affiliations:** ^1^School of Life Sciences, Institute of Aquatic Economic Animals, Guangdong Province Key Laboratory for Aquatic Economic Animals, Sun Yat-Sen University, Guangzhou, China; ^2^Biology Department, School of Life Sciences, Sun Yat-Sen University, Guangzhou, China

**Keywords:** ricefield eel *Monopterus albus*, isotocin, isotocin receptor, growth hormone, prolactin

## Abstract

The neurohypophyseal hormone oxytocin (Oxt) has been shown to stimulate prolactin (Prl) synthesis and release from the adenohypophysis in rats. However, little is known about the functional roles of Oxt-like neuropeptides in the adenohypophysis of non-mammalian vertebrates. In this study, cDNAs encoding ricefield eel oxytocin-like receptors (Oxtlr), namely isotocin (Ist) receptor 1 (Istr1) and 2 (Istr2), were isolated and specific antisera were generated, respectively. RT-PCR and Western blot analysis detected the presence of both Istr1 and Istr2 in the brain and pituitary, but differential expression in some peripheral tissues, including the liver and kidney, where only Istr1 was detected. In the pituitary, immunoreactive Istr1 and Istr2 were differentially distributed, with the former mainly in adenohypophyseal cell layers adjacent to the neurohypophysis, whereas the latter in peripheral areas of the adenohypophysis. Double immunofluorescent images showed that immunostaining of Istr1, but not Istr2 was localized to growth hormone (Gh) cells, but neither of them was expressed in Prl cells. Ist inhibited Gh release in primary pituitary cells of ricefield eels and increased Gh contents in the pituitary gland of ricefield eels at 6 h after *in vivo* administration. Ist inhibition of Gh release is probably mediated by cAMP, PKC/DAG, and IP3/Ca^2+^ pathways. In contrast, Ist did not affect either *prl* gene expression or Prl contents in primary pituitary cells. Results of this study demonstrated that Ist may not be involved in the regulation of Prl, but inhibit Gh release *via* Istr1 rather than Istr2 in ricefield eels, and provided evidence for the direct regulation of Gh cells by oxytocin-like neuropeptides in the pituitary of non-mammalian vertebrates.

## Introduction

In mammals, oxytocin (Oxt), an nonapeptide neurohormone, is produced in hypothalamic neurons and transported *via* axons to the neurohypophysis, from where Oxt is secreted into the systemic circulation (1). All mammals have a second neurohypophysial hormone, arginine vasopressin (AVP), which differs from Oxt by two amino acids and is believed to have arisen from a gene duplication event in evolution (2). The classical roles of Oxt are to regulate uterine contractility (3), and mediate milk ejection in response to suckling during lactation (4). Recently, accumulating evidence has established many other functions of Oxt, including electrolyte homeostasis, gastric motility, glucose homeostasis, adipogenesis, and osteogenesis in the periphery, and food reward, food choice, and satiety in the brain (1). In the pituitary of rat, the Oxt receptor (Oxtr) was shown to be localized to the anterior and posterior lobes (5). The concentrations of Oxt in the pituitary portal blood are 15–50 times higher than those in peripheral plasma (6). These lines of evidence suggest a possible role for Oxt in the regulation of the anterior pituitary. In support of this hypothesis, the release of Prl was shown to be stimulated by Oxt directly (7, 8). Oxt may also be involved in the regulation of GH (9, 10). However, there seems a controversy regarding the specific roles of Oxt on GH, with either inhibition (9) or stimulation (10) reported in rats. Furthermore, the information regarding to the regulation of the adenohypophysis by the neurohypophyseal neuropeptides in non-mammalian vertebrates is very limited.

Oxt-like and Avp-like neuropeptides are also identified in other vertebrates, including teleosts (11). Isotocin (Ist), a teleostean homolog of Oxt, differs from Oxt by one amino acid, with Ser instead of Gln on the fourth of the nonapeptide (11). In addition to the sequence conservation of the nonapeptide hormones, the mechanisms that regulate *ist* and *Oxt* genes have also been shown to be conserved during evolution (12). In contrast to mammals, two copies of Ist receptor genes, namely Ist receptor 1 (*istr1*) and *istr2*, were identified in most teleosts except stickleback (13). mRNA for Ist receptors was detected in the brain and some peripheral tissues, including the gill, intestine, spleen, liver, etc. (14, 15). In the forebrain of an African cichlid fish, the distribution of an Istr (Istr2) is widespread, with a pattern similar to those of Oxt-like neuropeptide receptors in other taxa (16), suggesting that the functions of the Ist signaling system may be conserved as compared to those of Oxt. Interestingly, the projection of immunoreactive Ist neurons was shown to innervate the hormone-producing cell populations in the pituitary of the sea bass (17) and terminate on Lh cells in the catfish (18). These lines of evidence suggest that Ist may also regulate the functions of the adenohypophysis in teleosts. However, the presence and physiological relevance of Ist receptors in the pituitary of teleost fish remains to be established.

The ricefield eel (*Monopterus albus*), a Synbranchiform fish of protogynous sex changing phenomenon, is becoming an important aquaculture species in China. Our previous results showed that Gh cells are located at the junction of the neurohypophysis and adenohypophysis in the pituitary of ricefield eels (19). In other teleosts including striped bass (*Morone saxatilis*) (20) and Atlantic halibut (*Hippoglossus hippoglossus* L.) (21), Gh cells were also found to be arranged in cords or multicellular layers adjacent to the neurohypophysis. These lines of evidence are suggestive of a possible functional relationship between Gh cells and neurohypophysis in teleosts. In this study, ricefield eel *istr1* and *istr2* cDNAs were isolated, and Istr1 and Istr2 antigens were prepared in *E. coli* and used to immunize rabbits to generate specific antisera against Istr1 and Istr2, respectively. Immunoreactive Istr1, but not Istr2 was shown to be localized to Gh cells, but neither of them was localized to Prl cells in the pituitary. Ist blocked basal Gh release, but not mRNA expression in the pituitary cells of ricefield eels possibly *via* cAMP, DAG/PKC, and IP3/Ca^2+^ pathways.

## Materials and Methods

### Animals and Tissues

The adult ricefield eels (body length 20–45 cm and body weight 20–45 g) used for this study were purchased from a local dealer in Guangzhou, Guangdong, China. Fish were sacrificed by decapitation, after which the pituitary gland and other tissues, including the brain, ovary, testis, muscle, spleen, pancreas, heart, liver, kidney, intestines, blood, eye, and bladder were dissected out and stored in a deep-freezer (−80°C) for tissue extracts, in RNA-later for isolation of RNA, or directly in ice-cold medium (the pituitary) for *in vitro* primary cell culture. The pituitary and gonadal tissues for histology and immunohistochemistry were fixed in Bouin’s solution for 24 h and stored in 70% ethanol until processing. The sex of ricefield eels could be assigned by visual inspection of the gonad at the time of dissection only in the female so that the phenotypic sex and gonadal developmental stages of experimental fish were further verified by histological examination. The ricefield eel embryos and larvae were obtained from Dazhong Breeding Co., Ltd., Jianyang, Sichuan, China. All procedures and investigations were reviewed and approved by the Center for Laboratory Animals of Sun Yat-sen University, and were performed in accordance with the Guiding Principles for the care and use of laboratory animals.

### Chemicals

Ricefield eel Ist was synthesized by ChinaPeptides Co., Ltd., (Shanghai, China). The purity of synthesized peptide is more than 95% (analyzed by HPLC) and its structure was verified by mass spectrometry. Rp-cAMPS was purchased from Santa Cruz (TX, USA), and Go6983 and U73122 from Selleckchem (TX, USA). All the other chemicals were obtained from Sigma Chemical Co. (St. Louis, MO, USA). The above reagents were carefully diluted in accordance with the respective manufacturer’s instructions to prepare the stock solution and stored at −80°C.

### Total RNA Extraction and Cloning of Ricefield Eel *istr1* and *istr2* cDNAs

Total RNA was extracted from ricefield eel tissues using TRIzol (Invitrogen, MA, USA) and quantified based on the absorbance at 260 nm. The A260/280 nm ratios for all RNA samples were between 1.9 and 2.0. The integrity of RNA was checked with agarose gel electrophoresis, and further verified by the successful amplification of *actb* (actin, beta; accession number AY647143.1).

To clone ricefield eel *istr1* and *istr2* cDNAs, the brain RNA was reverse transcribed with the RevertAid H Minus First Strand cDNA Synthesis Kit (Thermo Scientific, Waltham, MA, USA) according to the manufacturer’s instructions using the adapter primer AP. Then the initial fragments of *istr1* and *istr2* cDNAs were amplified from 1 µl of the brain cDNA by nested PCR. The primers were ISTR1-F1 and ISTR1-R1 for the first round and ISTR1-F1 and ISTR1-R2 for the second round of amplification for *istr1*, and ISTR2-F1 and ISTR2-R1 for the first round and ISTR2-F1 and ISTR2-R2 for the second round of amplification for *istr2*. These primers were degenerated and targeted to the nucleotide sequences in highly conserved regions of previously identified teleost *istr1* and *istr2* homologs, respectively. PCR was performed in a 25 µl final volume containing 2.5 µl 10 × Taq Buffer, 2.5 mM MgCl_2_, 0.2 mM dNTP, 0.4 µM of each primer, and 1.25 U Fermentas Taq DNA Polymerase (Fermentas, Waltham, MA, USA). After an initial 3 min denaturing step at 94°C, 38 cycles of amplification were performed with 30 s at 94°C, 30 s at 50 (first) or 52 (second)°C, and 120 s at 72°C, and then followed by a final extension for 10 min at 72°C using the TGRADIENT thermal cycler (Biometra GmbH, Goettingen, Germany). Target PCR products of about 600 bp were generated, which were confirmed to be *istr1* and *istr2*, respectively, by DNA sequencing and Blast analysis.

Then 3′ and 5′ ends of *istr1* and *istr2* cNDAs were obtained by the RACE method using nested PCR. For the 3′ ends, the primers were ISTR1-F2 and AUAP for the first round and ISTR1-F3 and AUAP for the second round of amplification of *istr1*, and ISTR2-F3 and AUAP for the first round and ISTR2-F4 and AUAP for the second round of amplification of *istr2*. For the 5′ ends, the primers were ISTR1-R3 and AAP for the first round and ISTR1-R4 and AUAP for the second round of amplification of *istr1*, and ISTR2-R3 and AAP for the first round and ISTR2-R4 and AUAP for the second round of amplification of *istr2*. The gene-specific primers were targeted to the non-conserved regions between *istr1* and *istr2*. The sequences of these primers were listed in Table S1 in Supplementary Material. The cycling conditions and processing of PCR products were the same as above.

### Sequence Analysis

Sequence alignment was performed by Clustalx1.83. Phylogenetic tree was constructed *via* MEGA 7.0. Homology analysis was performed with the MegAlign tool of DNAstar software. The oxytocin-like receptor (Oxtlr) amino acid sequences of representative species were downloaded from *NCBI*. Synteny analysis was performed using Ensembl database, and the collinear genes around the *oxtlr* genes were plotted by CorelDRAW X3 software.

### RT-PCR and Quantitative Real-Time PCR Analysis

Total RNA samples (1 µg) isolated from tissues were first treated with RNase-free DNase I (Thermo Scientific, Waltham, MA, USA) and reverse transcribed with random hexamer primers by using the RevertAid H Minus First Strand cDNA Synthesis Kit (Thermo Scientific, Waltham, MA, USA). Then 1 µl of cDNA was used for PCR detection of ricefield eel *istr1* and *istr2* mRNA in tissues, including the olfactory bulb, telencephalon, hypothalamus, optic tectum-thalamus, cerebellum, medulla oblongata, pituitary, ovary, testis, muscle, spleen, pancreas, heart, liver, kidney, intestines, blood, eye, and bladder. Parallel PCR for *actb* (actin, beta; accession number AY647143.1) was also performed to serve as the internal control. The primers were Istr1-SQ-F1 and Istr1-SQ-R1 for *istr1*, Istr2-SQ-F1 and Istr2-SQ-R1 for *istr2*, actb-QF1 and actb-QR1for *actb*. The upstream and downstream primers were targeted to different exons, respectively, and the sequences of primers were listed in Table S2 in Supplementary Material. All the gene-specific primers were searched and designed with the aid of the software Primer Premier 5.0. PCR was conducted for 38 cycles with 30 s at 94°C for denaturing, 30 s at 55°C for annealing, and 30 s at 72°C for extension. The PCR products were separated on a 1.5% agarose gel and stained with ethidium bromide (0.5 µg/ml). The gel image was captured with the G:BOX F3 Gel Documentation System (Syngene, Cambridge, United Kingdom). The specificity of PCR amplification was further confirmed by sequencing of PCR products. Three sets of tissue samples from male and female ricefield eels were analyzed and similar results were obtained for each sex, respectively. The representative electrophoretic images of one male and one female fish were presented.

Total RNA extracted from primary pituitary cells was reverse transcribed as above. Then 1 µl of cDNA template was used for real-time quantitative PCR analysis of *gh* (accession number AY265351.1) and *prl* (accession number MF996359) mRNA in primary cultured pituitary cells. The primers were GH-QF1 and GH-QR1 for *gh*, PRL-QF and PRL-QR for *prl*, actb-QF1 and actb-QR1 for *actb*, gapdh-QF and gapdh-QR for *gapdh* (accession number FJ873738.1), and hprt1-QF and hprt1-QR for *hprt1* (accession number DQ218476.1). The primers GH-QF1, GH-QR1, and PRL-QF are located at exon–exon junctions, and upstream and downstream primers for *actb, gapdh*, and *hprt1* are targeted to different exons, respectively. The nucleotide sequences of these primers were listed in Table S2 in Supplementary Material. The geometric mean expression levels of the latter three genes, *actb, gapdh*, and *hprt1*, were used to normalize the expression levels of the target genes. The real-time quantitative PCR was performed on the iCycler iQ5 (Bio-Rad) in a volume of 20 µl containing 0.2 µM of each primer, 10 µl of 2 × SYBR Green Master Mix (QPK-201, TOYOBO, Osaka, Japan), and 1 µl of cDNA template. The PCR cycling conditions were: 95°C for 3 min, 40 cycles of 95°C for 15 s, 58°C for 15 s, 72°C for 15 s, 82°C for 15 s for signal collection in each cycle. Data were produced and analyzed by iQ5 software. The specificity of PCR amplification was confirmed by melt-curve analysis, agarose gel electrophoresis, and sequencing of PCR products. All samples were run in duplicates and minus reverse transcriptase and no template controls were included in each assay.

The quantification of the mRNA expression level was performed using a standard curve with tenfold serial dilution of plasmid containing corresponding DNA fragments from 10^1^ to 10^8^ copies. The correlation coefficients and PCR efficiencies were not less than 0.98 and 95%, respectively. The copy numbers of *gh, prl*, and reference genes were calculated by iQ5 software (Bio-rad) based on the corresponding standard curves. The mRNA expression levels of *gh* and *prl* were presented as the copy number ratios to the geometric means of the three reference genes.

### Generation of Recombinant Ricefield Eel Istr1 and Istr2 Proteins and Respective Polyclonal Antisera

The cDNA sequences encoding segments of ricefield eel Istr1 (amino acid residues 334–394, Istr1 antigen) and Istr2 (amino acid residues 340–392, Istr2 antigen) were PCR amplified using gene-specific primer sets Istr1-F/Istr1-R and Istr2-F/Istr2-R, respectively. The PCR products were cloned into pET-32a *via Nco* I and *BamH* I sites and expressed in the host *E. coli* BL21 (*DE3*) as TRX fusion proteins by IPTG induction. The sequences of all primers used are listed in Table S2 in Supplementary Material. The recombinant Istr1 and Istr2 antigens were gel purified from inclusion bodies and used to immunize rabbits as previously reported (22). The anti-ricefield eel Prl antisera were also prepared in our lab and the detailed information was provided in the Supplemental Data in Supplementary Material.

The full open reading frames (ORFs) encoding ricefield eel Istr1 and/or Istr2 were also PCR amplified with primer sets pcDNA3.0-Istr1-F/pcDNA3.0-Istr1-R and pcDNA3.0-Istr2-F/pcDNA3.0-Istr2-R, respectively, and cloned into the expression vector pcDNA3.0. The expression constructs were transiently transfected into COS-7 cells and the cellular extracts containing recombinant full-length Istr1 and Istr2 proteins were prepared with RIPA lysis buffer (Beyotime), respectively, which was used as positive or negative controls in Western blot analysis for further examining the specificities of anti-Istr1 and anti-Istr2 antisera.

### Western Blot Analysis

The recombinant proteins or tissue homogenates of the brain, pituitary, gonad, spleen, liver, kidney, and intestine from female or male ricefield eels were separated on 12% SDS-PAGE gels and transferred to methanol-activated polyvinylidenefluoride membranes (Roche, Mannheim, Germany) by electroblotting. The membrane was then blocked with 5% nonfat milk powder in 0.01 M PBS (137 mM NaCl, 2.7 mM KCl, 10 mM Na_2_HPO_4_, 2 mM KH_2_PO_4_, pH 7.4) at 4°C overnight. The anti-Istr1 (1:1,000) or anti-Istr2 (1:1,000) antiserum was pre-adsorbed overnight at 4°C with extracts of *E. coli* BL21 (*DE3*) bacteria that were transformed with the empty vector pET32a and induced by IPTG. As negative controls for specificities, the anti-Istr1 and anti-Istr2 antisera were further pre-adsorbed with the extracts of COS-7 cells containing corresponding recombinant full-length Istr1 and Istr2 proteins, respectively. The blocked membrane was then incubated with the pre-adsorbed anti-Istr1 or anti-Istr2 (1:1,000), or mouse anti-Actb monoclonal antibody (1:2,000, 60008-1-Ig; ProteinTech Group, Inc., IL, USA) in blocking solution (5% nonfat milk powder in 10 mM PBS) at room temperature for 4 h, washed with PBS for 5 min three times, and incubated with horseradish peroxidase (HRP)-conjugated goat anti-mouse immunoglobulin G (IgG) (1:5,000; 115-035-003, Jackson ImmunoResearch Laboratories, Inc., PA, USA) for 1 hr at room temperature. After three 5 min final washes with PBS, the membranes were exposed to a chemiluminescence substrate (BeyoECL Plus kit, P0018, Beyotime, Shanghai, China) according to the manufacturer’s instructions.

### Immunohistochemistry

The Bouin-fixed pituitary gland (together with the brain) was embedded with paraffin and sectioned sagittally at 5 µm thickness. The pituitary sections were deparaffinized, hydrated, and incubated with 3% hydrogen peroxide solution to quench the endogenous peroxidase activity, followed by antigen retrieval in 10 mM citrate buffer (pH6.0) at 95°C for 15 min and blocking in 0.01 M PBS containing 10% normal goat serum for 30 min at room temperature. Then the sections were incubated with the primary rabbit anti-Istr1 (1:500), or anti-Istr2 (1:500), or anti-Prl antiserum (1:800) at 4°C overnight. After rinsing with PBS for 5 min three times, the sections were exposed to the secondary antibody (HRP-conjugated goat anti-mouse IgG, 1:500 dilution; 115-035-003, Jackson ImmunoResearch Laboratories, Inc., PA, USA) solution. After rinsing with PBS, the sections were developed with 3,3′-diaminobenzidine, mounted, examined with a Nikon Eclipse Ni-E microscope (Nikon, Japan), and digitally photographed with a Nikon DS-Ri2 digital camera. To confirm the specificity of the immunostaining, control sections were incubated with the primary antiserum (in its working solution) pre-adsorbed with an excess of corresponding recombinant Istr1, Istr2, or Prl antigen.

### Fluorescent Immunohistochemistry

Double-label fluorescent immunohistochemistry was performed as described previously (22). Briefly, deparaffinized sections of pituitary glands were boiled for 10 min in 10 mM sodium citrate buffer (pH 6.0) for antigen retrieval and blocked in 0.01 M PBS containing 10% normal goat serum for 30 min at room temperature. The blocked sections were then incubated in a primary antiserum mixture of rabbit anti-Istr1 (1:500) or anti-Istr2 antiserum (1:500) with mouse anti-Gh (19; 1:800) or mouse anti-Prl (1:800) antiserum for 12–16 h at 4°C. After rinsing with PBS for three times, the sections were exposed to the secondary antibody, a mixture of Cy3-labeled goat anti-mouse IgG (H + L) (1:500; catalog number A0521, Beyotime) and Alexa Fluor 488-labeled Goat Anti-Rabbit IgG (H + L) (1:500; catalog number A0423, Beyotime) for 1 hr at room temperature. After washing three times in 0.01 M PBS for 5 min, the sections were counterstained with 5 µg/ml 2-[4-amidinopheny]-6-indolecarbamidine (DAPI) (catalog number C1002, Beyotime), a nuclear counterstain, for 8 min at room temperature. After rinsing with PBS for 15 min, the sections were coverslipped using an antifade fluorescent mounting medium (catalog number P0126, Beyotime) and stored in the dark at 4°C. Fluorescent signals were visualized and photographed with a Nikon DS-Ri2 digital camera on a Nikon Eclipse Ni-E microscope, and the images were overlapped with the Nikon NIS-Elements BR software.

### ELISA for Ricefield Eel Gh

A competitive enzyme immunoassay for ricefield eel Gh was developed using the polyclonal anti-Gh antiserum (19) and Gh covalently coupled with biotin. The recombinant ricefield eel Gh was prepared as previously described (19) and further column purified with Zeba™ Spin Desalting Columns (Sigma Chemical Co.). The purified Gh was then labeled with biotin using EZ-Link™ Sulfo-NHS-LC-Biotin (Sigma Chemical Co.) according to the manufacturer’s instructions, and designated as Gh-biotin. Briefly, 96-well microtiter plates were coated with the rabbit polyclonal anti-Gh antiserum (1:1,000) in carbonate buffer (0.05 M sodium carbonate, pH 9.6) for 2 h at 37°C. The coated plates were extensively washed three times with 200 µl of 0.01 M PBS (137 mM NaCl, 2.7 mM KCl, 10 mM Na_2_HPO_4_, 2 mM KH_2_PO_4_, pH 7.4) containing 0.05% Tween 20 (PBST), and was then blocked with 1% BSA (Roche, Basel, Switzerland) for 2 h at 37°C. After washing twice with PBST, 50 µl of standard or sample solution and 50 µl of Gh-biotin (diluted at 1:2,000) were added to the wells. After overnight incubation at 4°C, the plates were then washed again as above and incubated with 100 µl per well of streptavidin-HRP conjugate (SA-HRP; Sigma Chemical Co.; diluted at 1:5,000 in 1% BSA) at 37°C for 1 h. After washing with PBST three times, 100 µl per well of TMB (3,3′,5,5′-Tetramethylbenzidine, Sigma Chemical Co.) was added and developed at 37°C for 15 min. The optical densities at 450 nm were measured using a Molecular Devices microplate reader (Epoch, Biotek, USA). The concentrations of unknown samples were calculated from a standard curve using a Log-logit plot. All measurements for standards and samples were made in duplicates. The validity of ricefield eel Gh ELISA for determining relative Gh contents in this study was analyzed by the correlation between the standard curve and dilution curves of homogenates of pituitary glands and culture media of pituitary cells, and cross reactivities with recombinant ricefield eel prolactin (Prl), somatolactin (Sl), luteinizing hormone beta subunit (Lhb), follicle-stimulating hormone beta subunit (Fshb), and thyroid-stimulating hormone beta subunit (Tshb), respectively.

### *In Vitro* Treatment of Cultured Ricefield Eel Pituitary Cells With Ist

The pituitary glands were dissected out from female ricefield eels and digested by trypsin (65 mg/ml; Gibco, MA, USA) at room temperature for 10 min. The dispersed pituitary cells were then seeded in 24-well plates (Nunc, Denmark) at approximately 1 × 10^6^ cells/mL per well with DMEM (Gibco, MA, USA) containing 10% FBS (Gibco, MA, USA) and cultured at 28°C with 5% CO_2_. After 16-h culture, the culture medium was removed and cells were incubated in DMEM without the addition of FBS in the presence or absence of Ist (4 wells per treatment) for the duration as indicated. At the end of treatment, culture medium was harvested for monitoring Gh release in each individual well, and cell lysate was prepared for measurement of cell content for Gh and Prl. The levels of Gh were quantified using the competitive ELISA for ricefield eel Gh, and the levels of Prl were analyzed by Western blot and further quantified with Gel-Pro analyzer software by applying the integrate optical density. The total production of Gh in individual wells was deduced pro rata based on the protein data for Gh release and cell content. In parallel experiments, total RNA was isolated from pituitary cells, and *gh* and *prl* mRNA expression levels were quantified with quantitative real-time PCR analysis. The *in vitro* experiments were repeated three or four times, and similar results were obtained.

### *In Vivo* Treatment of Ricefield Eels With Ist

A total of 132 female ricefield eels (body length 35–45 cm, body weight 40–50 g) were purchased from a local dealer in Guangzhou, Guangdong, P. R. China, and kept in twelve 50 l plastic tanks in laboratory under a natural photoperiod and room temperature in June 2017, with 11 fish each tank as a treatment group. The tank water was replaced on alternate days. After acclimatization for 3 days, ricefield eels received intraperitoneal injections of either Ist or 0.65% NaCl (vehicle control, 11 fish per treatment in a tank). For the time-course effects, Ist was administered at a dose of 0.1 µg/g body weight. The pituitary glands of ricefield eels were dissected out at 2, 6, and 12 h after injection (11 fish of one tank for each treatment at each sampling point), and homogenized individually in 200 µl of PBS. For the dose-dependent effects, Ist was administered at doses of 0.01 and 0.1 µg/g body weight, respectively. The pituitary glands of ricefield eels (11 fish of one tank for each treatment) were dissected out at 6 h after injection, and processed as above. The Gh content in the homogenate of each individual pituitary gland was analyzed with the competitive ELISA for ricefield eel Gh. The *in vivo* experiments were repeated twice, and similar results were obtained.

### Statistical Analysis

All data are presented as mean ± SEM. The significance of observed differences between groups was determined by one-way ANOVA followed by the Tukey multiple comparison test using the SPSS17.0 software (SPSS, Inc.). Statistical significance was set at *P* < 0.05.

## Results

### Sequence Analysis of Ricefield Eel Ist Receptors

**Figure 1:**
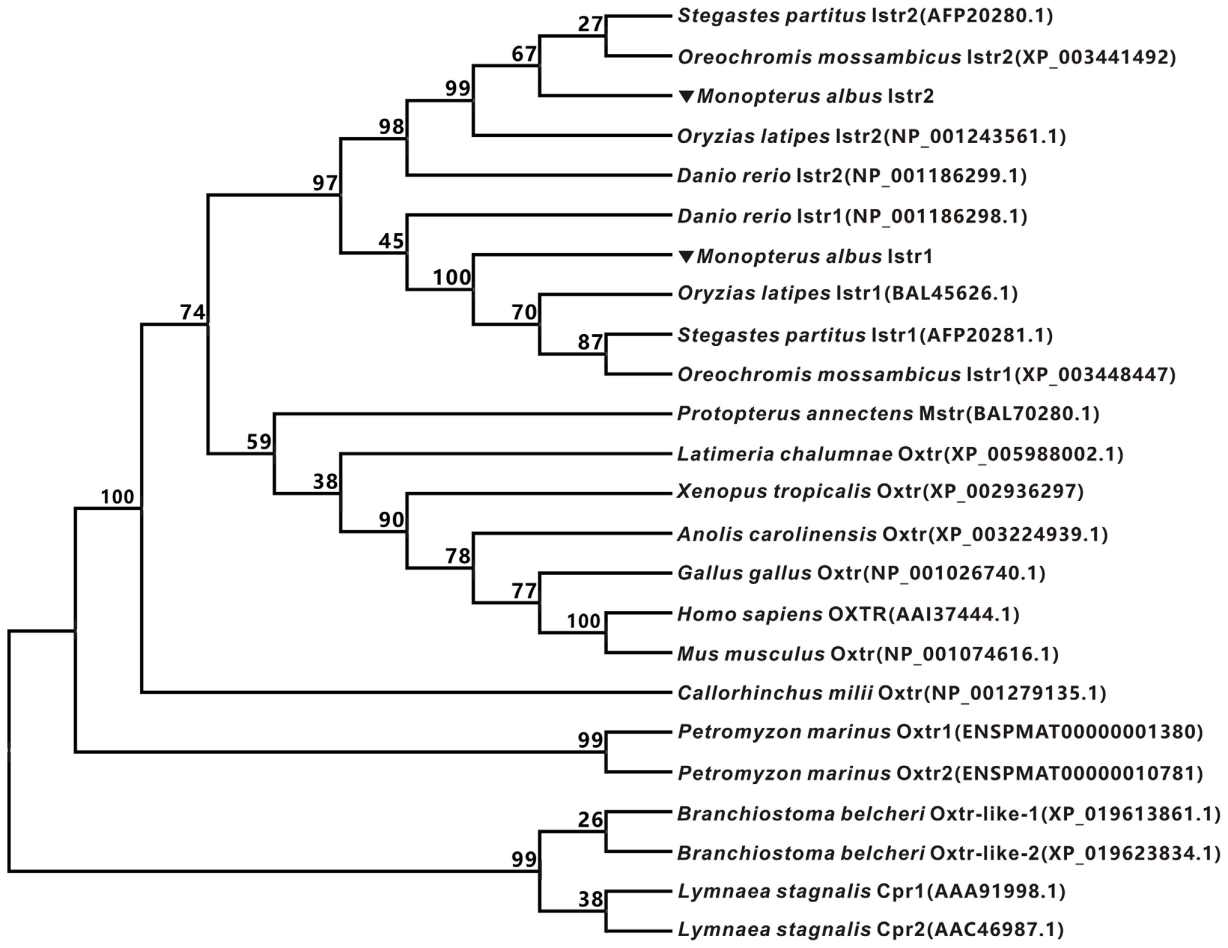
Phylogenetic analysis of oxytocin-like receptors of ricefield eel and other vertebrates. The phylogenetic tree was constructed based on an alignment of deduced amino acid sequences by Neighbor-Joining (NJ) method using Mega 7.0 software. The bootstrap values (%, 1,000 replicates) were given at each branch point. The protein sequences were downloaded from *Entrez* (NCBI).

**Figure 2:**
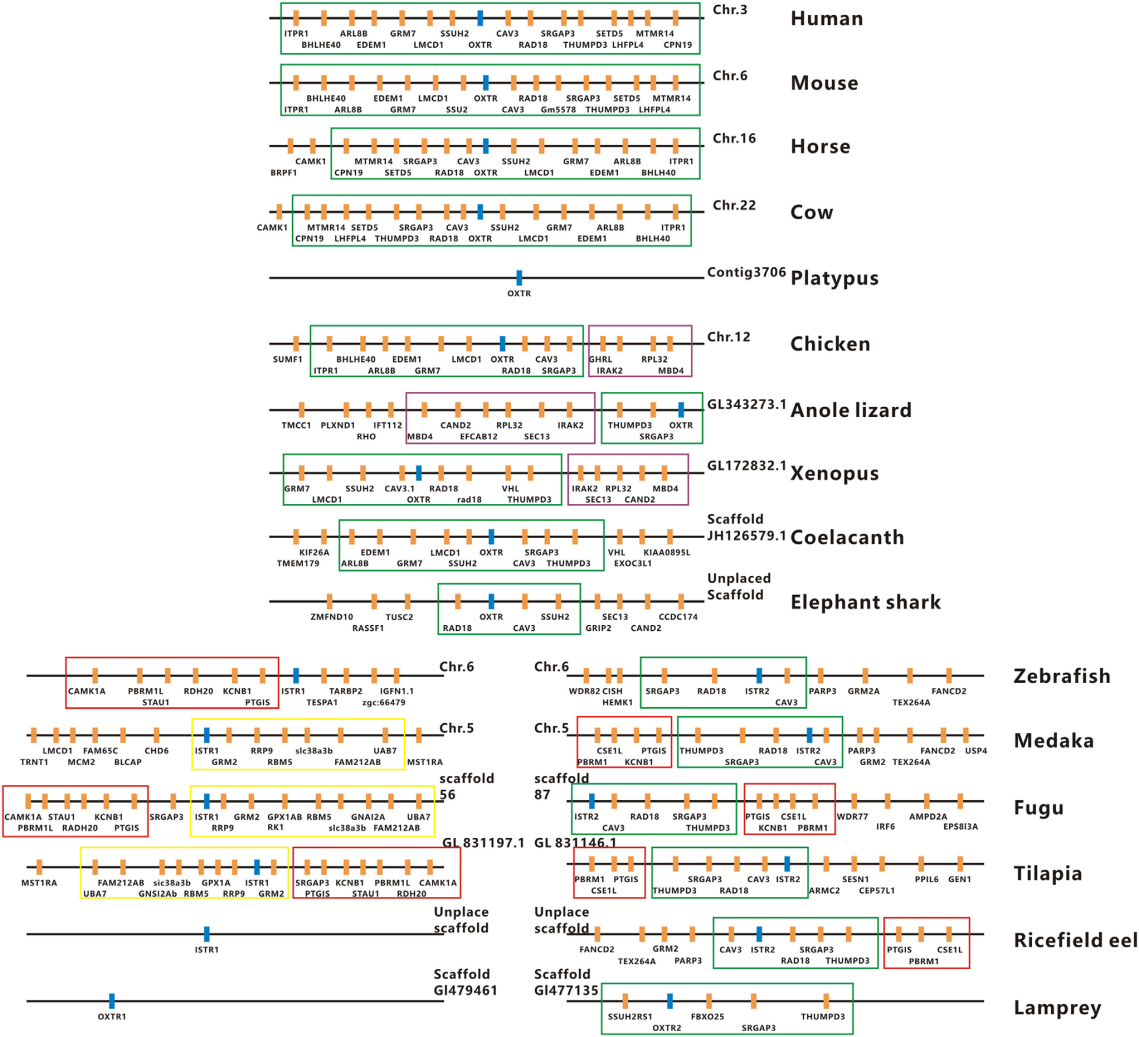
Physical maps of the genomic environments around *oxytocin-like receptor (oxtlr*) genes in vertebrates by synteny analysis. The *Oxtlr* genes are indicated by blue bars and other genes around *Oxtlr* genes by orange bars. The conserved DNA blocks are denoted by using different colored lines. The green line-boxed DNA blocks include almost the same gene content or gene order in vertebrates, and the purple line-boxed DNA blocks are present specifically in birds, reptiles, and amphibians. The red and yellow line-boxed DNA blocks are present specifically in teleosts. All the genomic data used were downloaded from http://asia.ensembl.org/index.html. Human, *Homo sapiens*; Mouse, *Mus musculus*; Horse, *Equus caballus*; Cow, *Bos taurus*; Platypus, *Ornithorhynchus anatinus*; Chicken, *Gallus gallus*; Anole lizard, *Anolis carolinensis*; Xenopus, *Xenopus tropicalis*; Coelacanth, *Latimeria chalumnae*; Elephant shark, *Callorhinchus milii*; Zebrafish, *Danio rerio*; Medaka, *Oryzias latipes*; Fugu, *Takifugu rubripes*; Tilapia, *Oreochromis mossambicus*; Ricefield eel, *Monopterus albus*; Lamprey, *Petromyzon marinus*.

The full-length cDNAs encoding two forms of ricefield eel Ist receptors were isolated, which were designated as *istr1* and *istr2*, respectively. The ricefield eel *istr1* cDNA (accession number MF996357) spans 2,476 bp and contains an ORF encoding a putative protein of 395 aa. The ricefield eel *istr2* cDNA (accession number MF996358) spans 2,278 bp and contains an ORF encoding a putative protein of 394 aa. Ricefield eel Istr1 shares 74.7, 78.1, 83.2, and 84.6% identities with its counterparts of zebrafish, medaka, tilapia, and bicolor damselfish, and Istr2 shares 84.8, 91.5, 94.4, and 95.5% identities with its counterparts of the above fish in the same order. Ricefield eel Istr1 and Istr2 share 74.3% identity, with relatively higher homologies in transmembrane domains (Figure S1 in Supplementary Material). In the phylogenetic tree (Figure 1) generated by the neighbor-joining method, teleost Istrs are categorized as Istr1 and Istr2 branches, into which ricefield eel Istr1 and Istr2 are clustered correspondingly. In contrast, mesotocin receptor (Mstr) of African lungfish and Oxtr of coelacanth fish were clustered with Oxtrs of tetrapods instead. Synteny analysis showed that genes around teleost *istr2*, but not *istr1* are highly conserved as compared to those around *oxtlr* genes in tetrapods (Figure 2).

### Tissue Patterns of Istr1 and Istr2 Expression in Ricefield Eels

**Figure 3:**
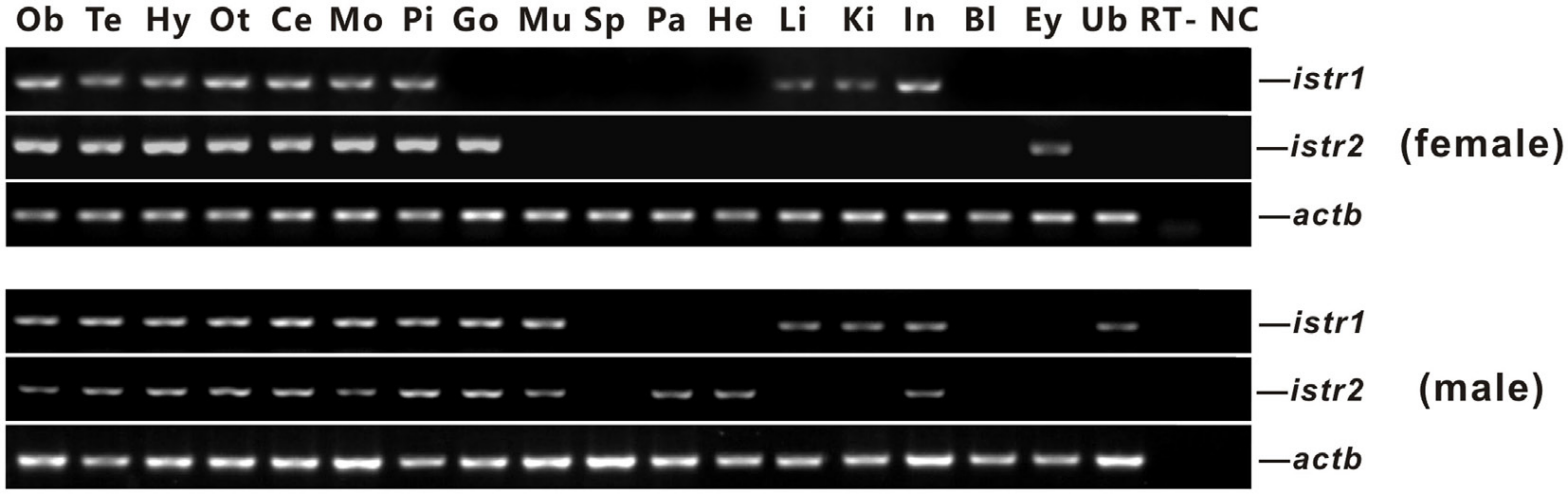
Tissue distributions of *isotocin (Ist) receptor 1* (*istr1*) and *istr2* mRNA in female and male ricefield eels as determined by RT-PCR. Abbreviations: Ob, olfactory bulb; Te, telencephalon; Hy, hypothalamus; Ot, optic tectum-thalamus; Ce, cerebellum; Mo, medulla oblongata; Pi, pituitary; Ov, ovary; Ts, testis; Mu, muscle; Sp, spleen; Pa, pancreas; He, heart; Li, liver; Ki, kidney; In, intestines; Bl, blood; Ey, eye; Ub, bladder; RT, RT minus (no addition of reverse transcriptase); NC, negative control (water used as template).

The mRNA expression of *istr1* and *istr2* in different tissues of male and female ricefield eels was examined with RT-PCR analysis (Figure 3). In the female, *istr1* mRNA was detected in the olfactory bulb, telencephalon, hypothalamus, optic tectum, cerebellum, medulla oblongata, pituitary, liver, kidney, and intestine, but undetectable in other tissues examined; *istr2* mRNA was detected in the olfactory bulb, telencephalon, hypothalamus, optic tectum, cerebellum, medulla oblongata, pituitary, ovary and eyes, but undetectable in other tissues examined. In the male, *istr1* mRNA was detected in the olfactory bulb, telencephalon, hypothalamus, optic tectum, cerebellum, medulla oblongata, pituitary, testis, muscle, liver, kidney, intestine, and urinary bladder, but undetectable in other tissues examined; *istr2* mRNA was detected in the olfactory bulb, telencephalon, hypothalamus, optic tectum, cerebellum, medulla oblongata, pituitary, testis, muscle, pancreas, and intestine, but undetectable in other tissues examined.

**Figure 4:**
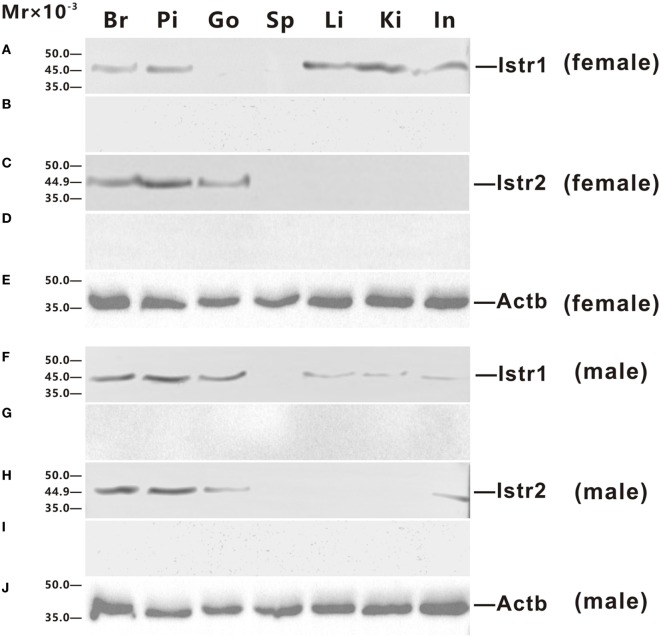
Western blot analysis of immunoreactive isotocin (Ist) receptor 1 (Istr1) and Istr2 in tissues of female **(A–E)** and male **(F–J)** ricefield eels. The tissue homogenates (500 µg) from the brain (Br), pituitary (Pi), gonad (Go), spleen (Sp), liver (Li), kindey (Ki), and intestine (In) were separated on 12% SDS-PAGE gels, transferred to polyvinylidenefluoride membranes, and then immunoreacted with the rabbit anti-Istr1 antiserum [1:1,000; **(A,F)**], or rabbit anti-Istr1 antiserum pre-absorbed by excessive recombinant full-length Istr1 **(B,G)**, or the rabbit anti- Istr2 antiserum [1:1,000; **(C,H)**], or rabbit anti-Istr2 antiserum pre-absorbed by excessive recombinant full-length Istr2 **(D,I)**, or mouse anti-Actb monoclonal antibody [1:2,000; catalog number: 60008-1-Ig; ProteinTech Group, Inc.; **(E,J)**]. The secondary antibody was 1:5,000 diluted horseradish peroxidase (HRP)-conjugated goat anti-mouse immunoglobulin G (IgG) (H + L) (catalog number 115-035-003; Jackson ImmunoResearch Laboratories) or HRP-conjugated goat anti-rabbit IgG (H + L) (catalog number 111-035-003, Jackson ImmunoResearch Laboratories, Inc., PE, USA). The blots were visualized using the BeyoECL Plus kit (Beyotime).

Polyclonal antisera against ricefield eel Istr1 and Istr2 were generated in this study, which were shown by Western blot analysis (Figure S2 in Supplementary Material) to specifically recognize ricefield eel Istr1 and Istr2, respectively. The expression of Istr1 and Istr2 in some tissues of male and female ricefield eels were further examined at the protein level with Western blot analysis (Figure 4). In the female, immunoreactive Istr1 was shown to be present in the brain, pituitary, liver, kidney, and intestine, but not in the ovary and spleen (Figure 4A); and immunoreactive Istr2 was shown to be present in the brain, pituitary, and ovary, but not in the spleen, liver, kidney, and intestine (Figure 4C). In the male, immunoreactive Istr1 was shown to be present in the brain, pituitary, testis, liver, kidney, and intestine, but not in the spleen (Figure 4F); and immunoreactive Istr2 was shown to be present in the brain, pituitary, testis, and intestine, but not in the spleen, liver, and kidney (Figure 4H). Pre-adsorption of the antisera with recombinant full-length Istr1 or Istr2 abolished immunoreactive signals in tissues (Figures 4B,D,G,I), further confirming the specificities of the antisera generated.

### Istr1 but Not Istr2 Is Co-Localized With Gh Cells but Neither of Them With Prl Cells in the Pituitary of Ricefield Eels

**Figure 5:**
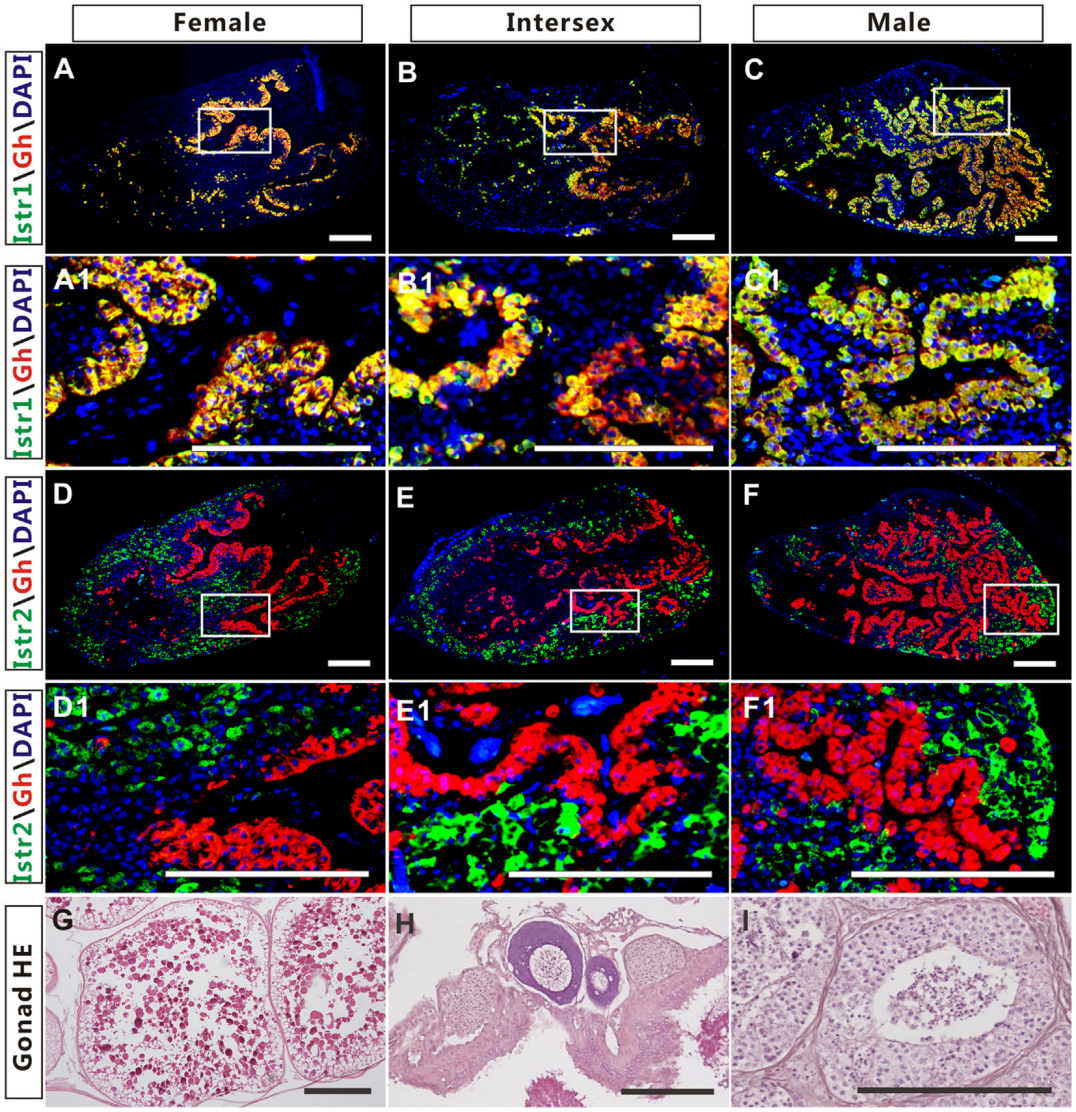
The co-localization of immunoreactive isotocin (Ist) receptor 1 (Istr1) (green) or Istr2 (green) with growth hormone (Gh) (red) in the pituitary of female, intersexual, and male ricefield eels. The rabbit antiserum against Istr1 (1:500) or Istr2 (1:500) and mouse antiserum against Gh (1:800) were used as primary antisera. The secondary antibodies were 1:500 diluted Alexa Fluor 488-labeled goat anti-rabbit immunoglobulin G (IgG) (H + L) for Istr1 and Istr2, and 1:500 diluted Cy3-labeled goat anti-mouse IgG (H + L) for Gh. DAPI was used to stain the nuclei blue. The images were observed and captured with a confocal microscope under the same conditions. **(A1–F1)** are higher magnification of **(A–F)**, respectively. The overlapping of the red with the green color generated a yellow color. **(G–I)**: HE-stained gonads of the experimental fish at female, intersexual, and male stages, respectively. Scale bar is 50 µm.

**Figure 6:**
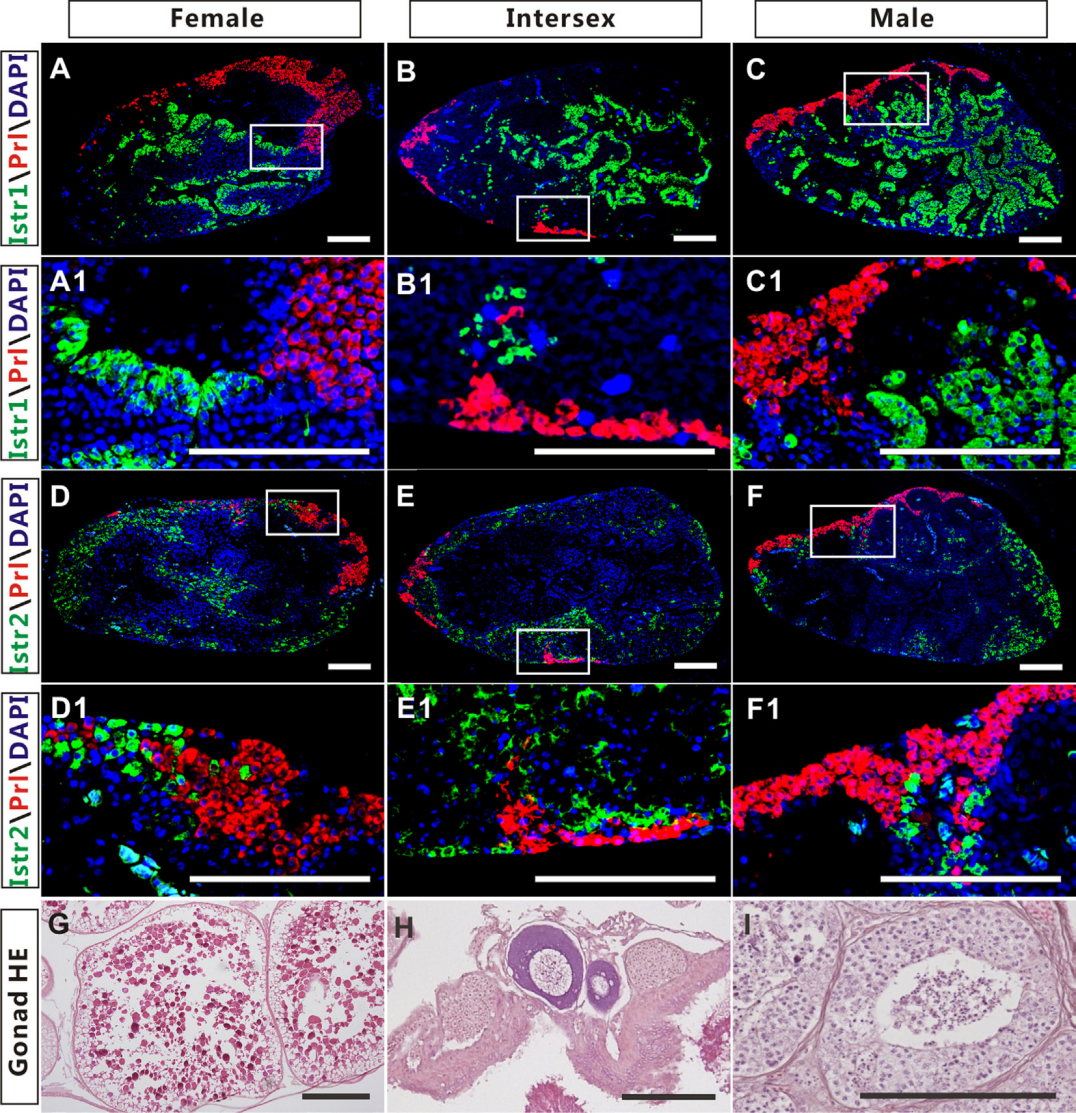
The cellular localization of immunoreactive isotocin (Ist) receptor 1 (Istr1) (green), Istr2 (green), and prolactin (Prl) (red) in the pituitary of female, intersexual, and male ricefield eels. The rabbit antiserum against Istr1 (1:500) or Istr2 (1:500) and mouse antiserum against Prl (1:800) were used as primary antisera. The secondary antibodies were Alexa Fluor 488-labeled goat anti-rabbit immunoglobulin G (H + L) for Istr1 and Istr2, and Cy3-labeled goat anti-mouse lgG (H + L) for Prl. DAPI was used to stain the nuclei blue. Sagittal sections of ricefield eel pituitaries were shown here with the rostral (anterior) to the left. **(A1–F1)** are higher magnification of **(A–F)**, respectively. **(G–I)**: HE-stained gonads of the experimental fish at female, intersexual, and male stages, respectively. Scale bar is 50 µm.

The high expression of Istr1 and Istr2 in the pituitary of female and male ricefield eels strongly suggests that Ist may be involved in the regulation of pituitary functions. The immunoreactive Istr1 and Istr2 signals in the pituitary of ricefield eels were shown to be differentially distributed by immunohistochemistry, with the former mainly located in the multicellular layers of the adenohypophysis adjacent to the neurohypophysis, and the latter in peripheral areas of the adenohypophysis (Figures S3A,C in Supplementary Material). Pre-absorption of anti-Istr1 and anti-Istr2 with recombinant Istr1 and Istr2 proteins abolished the immunostaining, respectively (Figures S3B,D in Supplementary Material), further confirming the specificities of the antisera generated. As the cellular localization of Istr1 in the pituitary is similar to that of Gh (19), the possible co-localization of Istr1 and Istr2 with Gh was examined with double fluorescent immunohistochemistry (Figure 5). In the pituitary of female, intersexual, and male fish, immunoreactive signals of Istr1 (Figures 5A–C and A1–C1), but not Istr2 (Figures 5D–F and D1–F1) were perfectly co-localized with Gh cells. The Istr1 immunostaining could be detected in Gh cells even at their first appearance in the embryos of 3 days post fertilization during ontogeny (Figure S4 in Supplementary Material). We further examined the possible co-localization of immunoreactive Istr1 and Istr2 with Prl (Figure 6) using the highly specific anti-ricefield eel Prl antiserum (Figure S5 in Supplementary Material). Neither Istr1 (Figures 6A–C and A1–C1) nor Istr2 (Figures 6D–F and D1–F1) immunostaining was detected in Prl cells in the pituitary of female, intersexual, and male ricefield eels.

### Ist Blocks Gh Release From Pituitary Cells of Female Ricefield Eels

**Figure 7:**
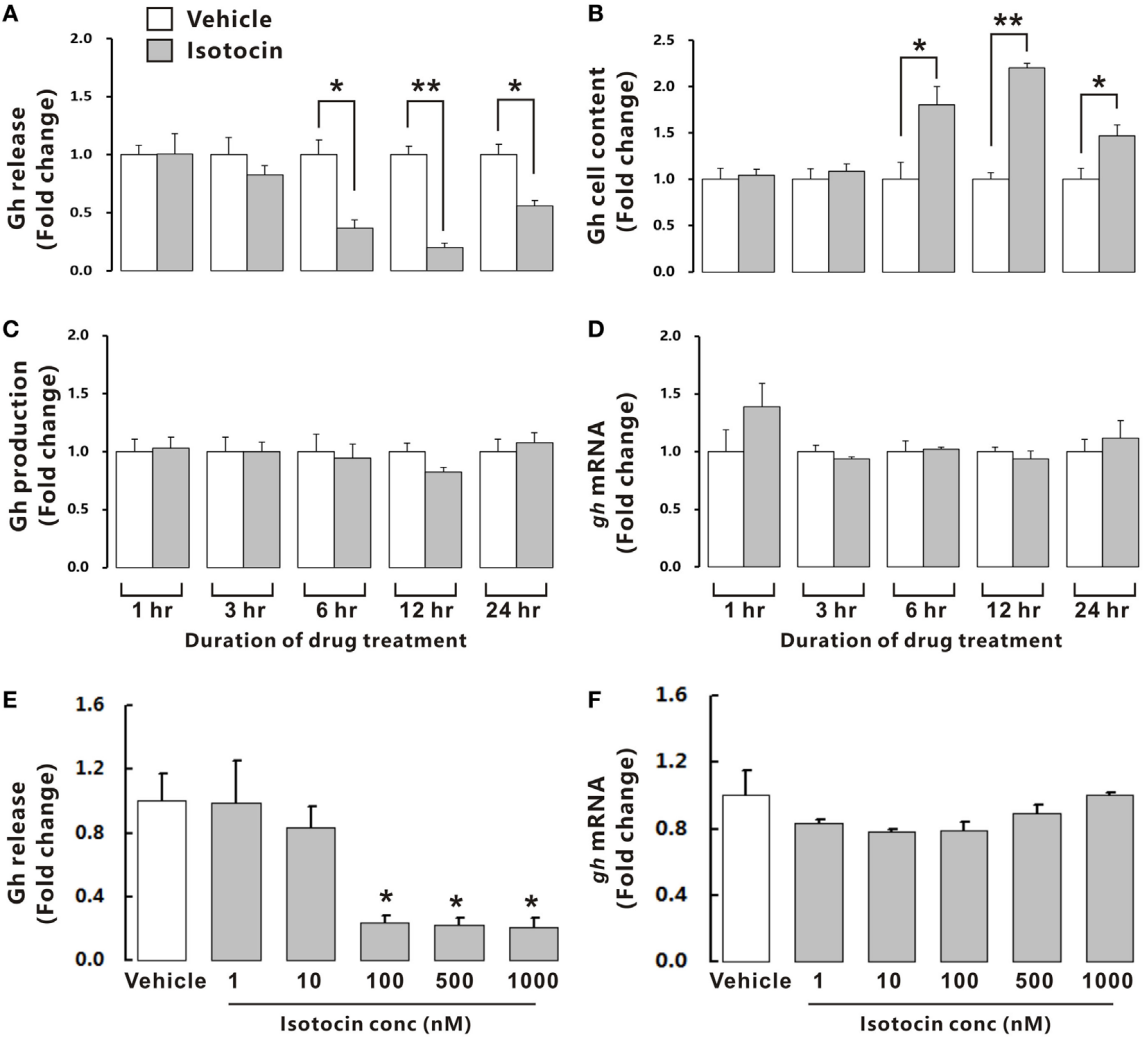
Effects of isotocin (Ist) on growth hormone (Gh) synthesis and release in primary pituitary cells of female ricefield eels. Time course of Ist (100 nM) on **(A)** Gh release, **(B)** cell content, **(C)** total production, and **(D)** *gh* mRNA in ricefield eel pituitary cells. **(E,F)** Dose-dependence of 12-h treatment with increasing levels of Ist (1–1,000 nM) on Gh release and *gh* mRNA, respectively. After drug treatment, culture medium was harvested for measurement of Gh release, and cell lysate was prepared for monitoring Gh contents in pituitary cells. In parallel experiments, total RNA was isolated for real-time PCR analysis of *gh* mRNA. Data were expressed as fold change relative to the corresponding vehicle control. Bars represent mean ± SEM of pooled results from four separate experiments (*n* = 4). *P < 0.05; **P < 0.01 between the indicated groups or vs. the vehicle control **(E)**.

**Figure 8:**
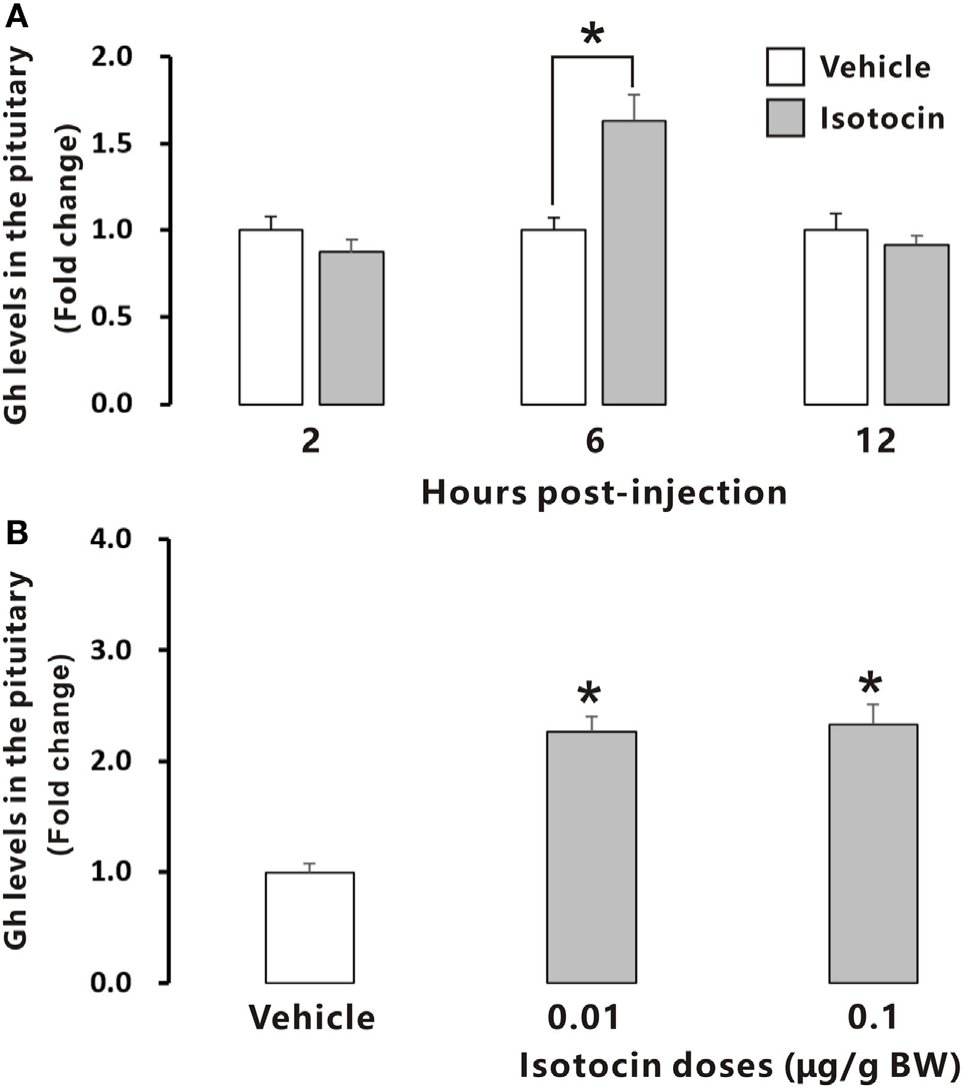
Relative growth hormone (Gh) levels in the pituitary of female ricefield eels after intraperitoneal injection of isotocin (Ist) or 0.65% NaCl (Vehicle control). **(A)** The time-course effects of Ist (0.1 µg/g BW) at 2, 6, and 12 h after injection; **(B)** dose-dependent effects of Ist (0.01 and 0.1 µg/g BW) at 6 h after injection; BW, body weight. Data were expressed as fold change relative to the corresponding vehicle control. Bars represent mean ± SEM (*n* = 11). *P < 0.05 vs. the corresponding control.

To examine the possible involvement of Ist in the regulation of Gh release, ricefield eel Gh Elisa was established and validated for determining relative Gh contents in homogenates of pituitary glands and culture media of pituitary cells (Figures S6 and S7 in Supplementary Material). The ricefield eel Gh Elisa has the detection range from 3.1 to 6,400 ng/mL, with the intraassay CV <6.6% and the interassay CV <8.4%. In primary pituitary cells of female ricefield eels cultured *in vitro*, basal Gh levels in the culture medium ranged from about 70 to 120 ng/mL. Results of time-course experiments showed that the amounts of Gh released in Ist (100 nM)-treated pituitary cells were significantly lower than those of control pituitary cells at 6, 12, and 24 h of incubation (Figure 7A). In contrast, the cellular Gh contents of Ist-treated (100 nM) pituitary cells were significantly higher than those of the control pituitary cells at 6, 12, and 24 h of incubation (Figure 7B). The total amounts of Gh production (Figure 7C) and *gh* mRNA levels (Figure 7D) were not significantly different between Ist (100 nM)-treated and control pituitary cells. Results of dose-dependent studies showed that 12-h incubation with increasing levels of Ist (1–1,000 nM) also blocked Gh release in a dose-related fashion (Figure 7E), but *gh* transcript levels were not significantly altered (Figure 7F). In the pituitary of female ricefield eels treated with Ist (0.01 and 0.1 µg/g BW), the Gh content was significantly increased as compared to the vehicle control after treatment for 6 h (Figure 8).

**Figure 9:**
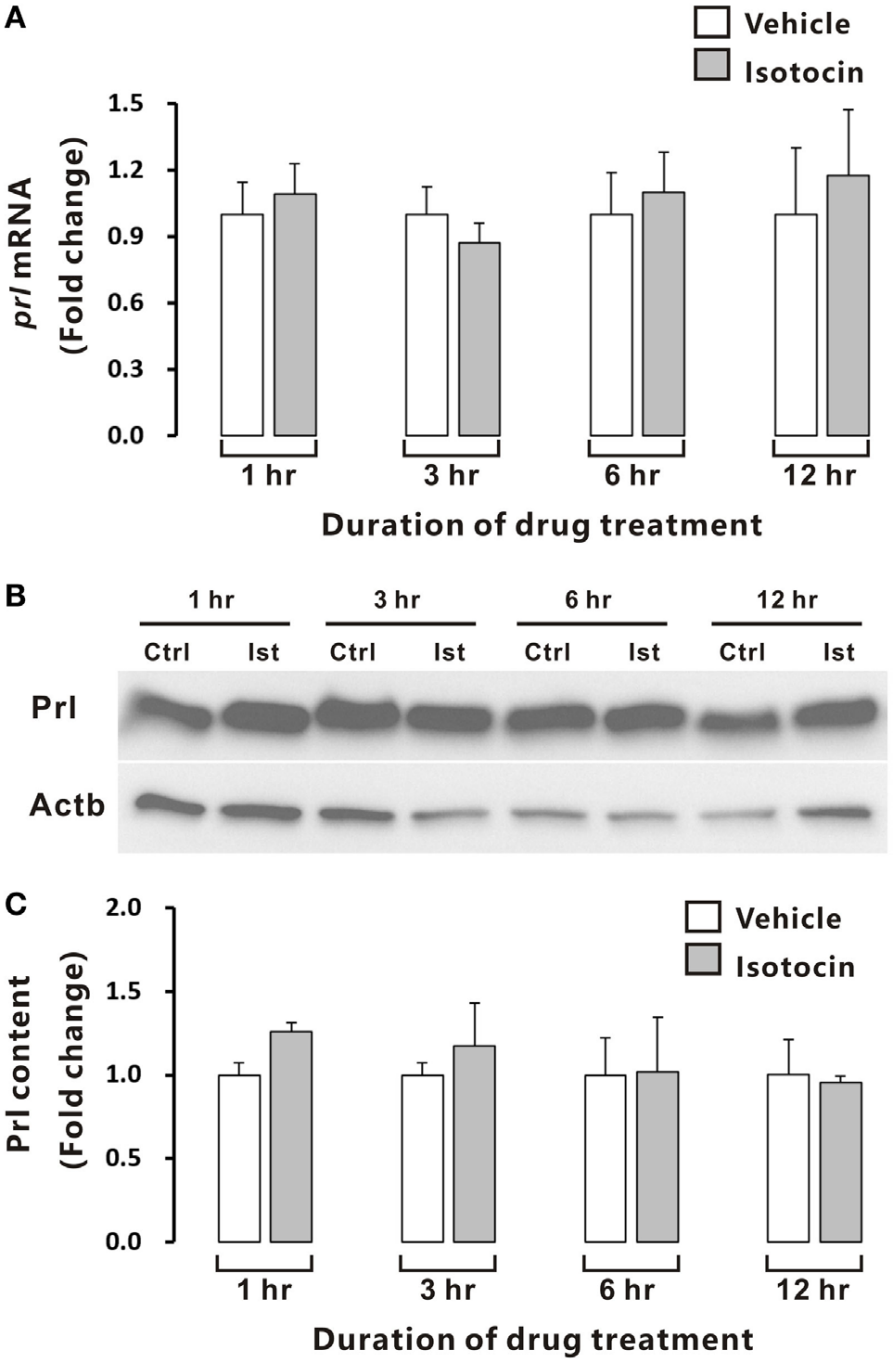
Effects of isotocin (Ist) (100 nM) on *prl* gene expression and prolactin (Prl) contents in ricefield eel pituitary cells. The primary cultured pituitary cells were pre-incubated for 12 h before treating with Ist (100 nM) for 1, 3, 6, and 12 h. *prl* mRNA levels **(A)** were analyzed by real-time PCR, and Prl contents were examined with Western blot analysis **(B)** and further quantified **(C)** with Gel-Pro analyzer software by applying the integrate optical density, and normalized by those of Actb. Data were expressed as fold change relative to the corresponding control. Bars represent mean ± SEM of pooled results from three separate experiments (*n* = 3). Ctrl, vehicle control.

In contrast, quantitative PCR and Western blot analysis showed that Ist did not affect either *prl* gene expression (Figure 9A) or Prl contents (Figures 9B,C) in the cultured pituitary cells of female ricefield eels.

### Signaling Pathways Involved in Ist Inhibition of Gh Release in Ricefield Eel Pituitary Cells

**Figure 10:**
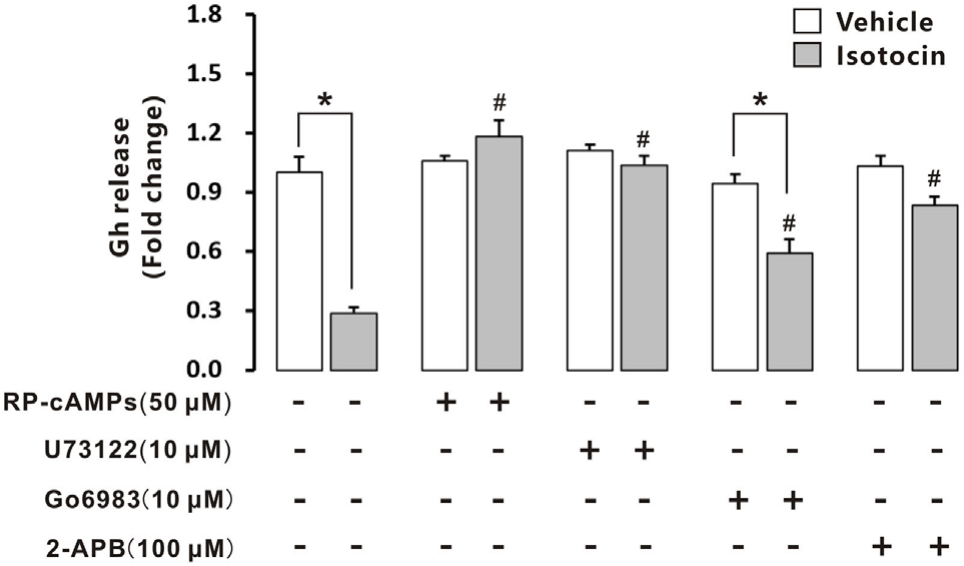
Effects of intracellular signaling pathway inhibitors on isotocin (Ist) inhibition of growth hormone (Gh) release. The primary cultured pituitary cells were pre-incubated for 12 h before being treated with Ist (100 nM) in the presence or absence of inhibitors Rp-cAMPS (50 µM), Go6983 (10 µM), U73122 (10 µM), or 2-APB (100 µM) for 12 h, respectively. After drug treatment, the culture medium was harvested for measurement of Gh release by competitive ELISA. Data were expressed as fold change relative to the vehicle control in the absence of inhibitors. Bars represent mean ± SEM of pooled results from four separate experiments (*n* = 4). *P < 0.05 between the indicated groups, ^#^*P* < 0.05 vs. the group treated only with isotocin (one-way ANOVA followed by the turkey test).

Isotocin (100 nM) could significantly inhibit Gh release in cultured primary pituitary cells of female ricefield eels pituitary cells after incubation for 12 h (Figure 10). When RP-cAMPs (50 µM; a PKA inhibitor) or U73122 (10 µM; a PLC inhibitor) was added to the incubation, the inhibition of Gh release by Ist was completely abolished. Addition of Go6983 (10 µM; a PKC inhibitor) increased Gh release of Ist-treated pituitary cells to about 70% of the vehicle control, which is significantly higher than that of Ist-treated cells, but significantly lower than that of Go6983-treated cells. Addition of 2-APB (100 µM; an IP3R inhibitor) increased Gh release of Ist-treated pituitary cells to about 80% of the vehicle control, which is significantly higher than that of Ist-treated cells. Treatment with RP-cAMPs, U73122, Go6983, or 2-APB alone did not affect Gh release in the cultured pituitary cells. In addition, Ist increased cAMP concentrations (Figure S8A in Supplementary Material) and induced a transient elevation of intracellular calcium concentrations (Figure S8B in Supplementary Material) in primary pituitary cells of female ricefield eels.

## Discussion

It has been established that a single copy of oxytocin receptor (Oxtr) is present in mammals (7) and chicken (13). In contrast, our present study identified two forms of receptors for isotocin (Ist; an oxytocin-like neuropeptide in teleosts), namely Ist receptor 1 (Istr1) and Ist receptor 2 (Istr2), in ricefield eels. Similarly, two forms of Ist receptors have also been identified in the genome database of zebrafish, medaka, and tilapia (13). Phylogenetic analysis (Figure 1) categorized ricefield eel Istr1 and Istr2 with their counterparts of other teleosts, respectively. It is of interest to note that only one Oxtlr was identified in Sarcopterygii including African lungfish and Coelacanth, which is clustered with Oxtlr molecules in tetrapods as a monophyletic group in the phylogenetic tree. These lines of evidence suggest that the duplication of Ist receptors may occur only in Actinopterygii. *Istr1* and *istr2* genes are on the same chromosome in zebrafish and medaka (13), indicating that the two forms of *istr* genes may arise through tandem duplication during early stage of the Actinopterygii evolution. Homology analysis showed that ricefield eel Istr2 shared higher identities with its counterpart in teleosts and mammalian Oxtrs than ricefield eel Istr1. Moreover, genes around *istr2* but not *istr1* in teleosts showed high synteny with those around mammalian Oxtr and Oxtlr in other vertebrates. These lines of evidence suggest that after duplication, teleost Istr2 may be under more stringent selection pressure than Istr1, and Istr2 may have conserved functions as those of Oxtr, whereas Istr1 may have gained new functions during Actinopterygii evolution.

The distribution of Oxtlr has been shown to be widespread throughout the brain, including Oxtrs in mammals (5, 23), Mstrs in frogs (24), and Istrs in teleosts, such as *Sparus aurata* (Istr1) (15), *Catostomus commersoni* (Istr2) (14), and *Astatotilapia burtoni* (Istr2) (16). In agreement, mRNA for ricefield eel *istr1* and *istr2* were detected in all the brain regions by RT-PCR, and the expression of Istr1 and Istr2 in the brain of ricefield eels was further confirmed by Western blot analysis with homologous specific antiserum, respectively. The Oxt system is an evolutionarily conserved neuroendocrine mechanism that regulates reproductive and social behaviors across divergent taxa (25), such as pair bonding in a socially monogamous songbird (26) and parental care in rats (27). It has been demonstrated that the neural distribution of Oxtrs may underlie variations in social behavior in voles (28). Thus the distribution of Istr1 and Istr2 in the brain of ricefield eels would be worth of further study to shed light on their neuroendocrine roles, which is underway in our laboratory. The ricefield eel has unique behaviors of building bubble nest for hatching fertilized eggs and guarding the young after hatching till they are on their own (29). This kind of parental care behaviors, which is lacking in models like zebrafish, makes this species a unique model for studying oxytocin-like neuropeptides and their action mechanisms in social behaviors of vertebrates, including humans.

It is not clear whether there is any sub-functionalization between Istr1 and Istr2 in teleosts (15). Our present study showed that in some peripheral tissues of ricefield eels, differential expression for Istr1 and Istr2 were observed at mRNA and/or protein levels. In the female, the liver, kidney, and intestine expressed *istr1*, but not *istr2*, whereas the ovary and eye expressed *istr2*, but not *istr1*. In the male, the liver, kidney, and bladder expressed *istr1*, but not *istr2*, whereas the pancreas and heart expressed *istr2* but not *istr1*. These lines of evidence were the first to suggest possible subfunctionalization and/or neofunctionalization of Istr1 and Istr2 during the evolution of Actinopterygian lineage. In rats, the kidney expresses Oxtrs, and Oxt injected intraperitoneally caused dose-dependent increases in urinary osmolality (30). In teleosts, the kidney is also one of the organs involved in osmoregulation. Administration of Ist (1 pg–1 ng/kg body weight) reduced urine production in chronically cannulated European eels adapted to FW, and high doses (more than 10 ng/kg body weight) resulted in diuresis (31). The above results suggest that Ist may also regulate renal osmoregulation, probably *via* Istr1, but not Istr2 in ricefield eels. Sexual dimorphic expression of *istr1* and *istr2* was also observed in some peripheral tissues of ricefield eels, with *istr1* expression detected in the gonad, muscle, and bladder of only male fish, and *istr2* expression detected in the eye of only female fish and in the muscle, pancreas, and heart of only male fish. These results are suggestive of differential sexual functions and/or regulation of *istr1* and *istr2* in these tissues of ricefield eels. Although the expression of *istr1* was not detected in the pituitary of the gilthead sea bream (15), our present study revealed expression of both *istr1* and *istr2* mRNA and proteins in the pituitary of both female and male fish, suggesting that Ist signals may play roles in the regulation of pituitary functions in ricefield eels.

The expression of Oxtlr in the pituitary was also reported in other vertebrates, including Oxtr in rats (5, 32) and Mstr in frogs (24). In the pituitary of rats, Oxtr expression was found to be restricted to lactotrophs in an early study (7), but later on, it was also found to be expressed in somatotrophs and gonadotrophs (10). In non-mammalian vertebrates, however, the information about the cellular localization of Oxt-like receptors in the pituitary is still limited. In the frog pituitary, Mstr was shown to be expressed in the distal lobe (24), where Gh cells are presumably localized (33). Using homologous-specific antisera, our study showed that immunoreactive Istr1 and Istr2 were localized to different areas in the pituitary of ricefield eels, further suggesting possible subfunctionalization/neofunctionalization of ricefield eel Istr1 and Istr2. Similar to Oxtr expression in Gh cells of rat pituitary glands (10), one Ist receptor, Istr1 but not Istr2, was shown to be localized to Gh cells, suggesting that Ist may bind to Istr1 and regulate the function of Gh cells in the pituitary of ricefield eels. Moreover, Istr1 expression in Gh cells could be detected along with the appearance of Gh cells during ontogeny in the pituitary, indicating that regulation of Gh cells by Ist signals may be established at the differentiation of Gh cells in the pituitary glands. In contrast to the cellular localization of Oxtr in Prl cells in the pituitary of rats (7), Prl cells in the pituitary of ricefield eels showed no expression of either Istr1 or Istr2. In agreement, Ist did not affect either *prl* gene expression or Prl release in primary pituitary cells. These lines of evidence suggest that Ist signals may not regulate the function of Prl cells in the pituitary of ricefield eels. In conscious White Leghorn cockerels, it has also been shown that continuous infusion of 0.1, 1.0, and 10.0 mU/min/kg body weight of mesotocin had no effect on plasma Prl levels (34). Prl is best known for its role in stimulating the mammary glands to produce milk (lactation) in mammals (35). In lactating (breastfeeding) mothers, Oxt acts as the mammary glands, causing milk to be “let down” into subareolar sinuses (3). Thus, it is likely that Oxt stimulation of Prl release in mammals may be a newly acquired feature of oxytocin-like neuropeptides with the advent of breast feeding in mammals.

Growth hormone (Gh), a single chain polypeptide hormone secreted from Gh cells in the anterior pituitary, plays important roles in the growth and development of vertebrates. Previous studies has established that the secretion of Gh is regulated by many neuroendocrine factors produced by the brain (especially the hypothalamus), including stimulators like Gh-releasing hormone (36) and ghrelin (37), and inhibitors like somatostatin (SS) (36) and neuropeptide W (38). Recently, it has been shown that Oxt stimulates GH release possibly directly *via* Oxtr in the pituitary of rats (10), adding a new player in the list of neuroendocrine factors regulating Gh cell functions. In striking contrast, Ist, an oxotocin-like neuropeptide in teleosts, inhibits basal Gh release, but has no effects on *gh* mRNA expression and Gh production in primary pituitary cells of ricefield eels. Moreover, administration of Ist *in vivo* increased Gh contents in the pituitary of female ricefield eels, which is in line with the inhibitory effects of Ist on Gh release as revealed *in vitro*. These lines of evidence suggest that Ist signals are inhibitory to Gh release in the pituitary of ricefield eels. In the tilapia, a teleost belonging to Percomorpha as the ricefield eel, intraperitoneal injection of Ist decreased serum Gh levels at 6 h after injection (Figure S9 in Supplementary Material). However, a recent report showed that Ist stimulated Gh release from primary pituitary cells of goldfish (39), a teleost belonging to Cyprinomorpha. The discrepancy in the regulation of Gh release by Ist between ricefield eels and goldfish is probably related to species differences. Nevertheless, our present study represents the first report demonstrating a novel functional role for Oxt-like peptides in the pituitary of non-mammalian vertebrates.

Oxytocin receptors are functionally coupled to Gq/11α class GTP binding proteins, which by stimulating together with Gβγ the activity of phospholipase C-β isoforms, leads to the generation of inositol trisphosphate and 1,2-diacylglycerol, with the former triggering Ca^2+^ release from intracellular stores, whereas the latter stimulating protein kinase C (40). Ca^2+^ has been shown to play an important role in Oxtr-mediated intracellular signal transduction in rat pituitary Gh (10), Prl (8, 41), and Acth (42, 43) cells. In primary pituitary cells of ricefield eels, the inhibition of Gh release by Ist was completely attenuated by U73122 (a PLC inhibitor), and partially by Go6983 (a PKC inhibitor), or 2-APB (an IP3R inhibitor). Furthermore, Ist induced a transient elevation of intracellular calcium concentrations in primary pituitary cells of ricefield eels. These results suggest that both DAG/PKC and IP3/Ca^2+^ pathways are involved in the intracellular signal transduction mediating Ist inhibition on Gh release, which is in agreement with intracellular signal transduction of Oxt receptors revealed in the above studies. In addition, RP-cAMPS, a PKA inhibitor, also completely attenuated Ist inhibition on Gh release. Moreover, Ist increased cAMP concentrations in primary pituitary cells of ricefield eel. These lines of evidence suggest that PKA/cAMP pathway may also be involved in the intracellular signal transduction mediating Ist inhibition on Gh release in the pituitary of ricefield eels.

In conclusion, our present study identified two forms of Ist receptors, Istr1 and Istr2, in ricefield eels, and generated specific antisera against Istr1 and Istr2, respectively. In the pituitary of ricefield eels, Istr1 but not Istr2 was shown to be localized to Gh cells, whereas neither of them was expressed in Prl cells. Ist blocks the basal Gh release from ricefield eel pituitary cells possibly *via* PKA/cAMP, DAG/PKC, and IP3/Ca^2+^ pathways. Taken together, results of the present study showed that Ist may act as an inhibitor of Gh release in ricefield eels, and provided evidence for the direct regulation of Gh cells by oxytocin-like peptides in the pituitary of non-mammalian vertebrates. The physiological relevance of Gh inhibition by Ist in ricefield eels is worth of further study.

## Ethics Statement

All procedures and investigations were reviewed and approved by the Center for Laboratory Animals of Sun Yat-sen University, and were performed in accordance with the Guiding Principles for the care and use of laboratory animals.

## Author Contributions

WZ and LZ conceived and designed the research. WY, NZ, BS, and SZ performed the experiments. WY, NZ, and WZ analyzed data. WY, LZ, and WZ wrote the manuscript.

## Conflict of Interest Statement

The authors declare that the research was conducted in the absence of any commercial or financial relationships that could be construed as a potential conflict of interest.
