# Supplementary material for: Isotocin Regulates Growth Hormone but Not Prolactin Release From the Pituitary of Ricefield Eels
Source: Front Endocrinol (Lausanne). 2018 Apr 12;9:166. doi: 10.3389/fendo.2018.00166 (PMC5906535; doi:10.3389/fendo.2018.00166)
Supplement: Supplementary file 1 [file Table_1.DOCX]

**Supplemental Table 1.** Sequences of oligonucleotide primers used in cloning of *istr1* and *istr2*.

| Primer Name | Sequence (5’→3’) |
| --- | --- |
| **ISTR1-F1**  **ISTR1-F2**  **ISTR1-F3**  **ISTR1-R1**  **ISTR1-R2**  **ISTR1-R3**  **ISTR1-R4**  **ISTR2-F1**  **ISTR2-F2**  **ISTR2-F3**  **ISTR2-F4**  **ISTR2-R1**  **ISTR2-R2**  **ISTR2-R3**  **ISTR2-R4**  **AP**  **AAP**  **AUAP**  **AP1**  **AP2** | AAAGTGGARGTKRCYGTYCTGGT  GTCGTGGGCATGTTTGCATCTACT  TTACTTGCTCCCAGTGGCGATTT  CATBGMRATGATGAAGGCCATGTC  GCTGCTSACRCGNGAVAGBGGWTG  TGGTAAAACCTGAAAGATTGCAACG  AATATCAGGCTAAGCAACCACGAGG  ATGAARCACYTVAGCATYGCNGA  TGGGAYATYACNTTYMGSTTYTA  ATCGGGAGTGTATGACTGCTGG  AAAACCTCCAAAGGCAACACG  TARATCCABGGGTTRCAGCAGCT  CCACATCTGVACDRAGAARAA  TGGAGGCGAACATCCCAACGAC  AATGAACGAAGGGGCTGGCAAATAG  GGCCACGCGTCGACTAGTAC(T)16  GGCCACGCGTCGACTAGTAC(G)10  GGCCACGCGTCGACTAGTAC  GTAATACGACTCACTATAGGGC  ACTATAGGGCACGCGTGGT |

F: sense primer; R: antisense primer. All the primers are targeted to genes in the ricefield eel.
