# Supplementary material for: Isotocin Regulates Growth Hormone but Not Prolactin Release From the Pituitary of Ricefield Eels
Source: Front Endocrinol (Lausanne). 2018 Apr 12;9:166. doi: 10.3389/fendo.2018.00166 (PMC5906535; doi:10.3389/fendo.2018.00166)
Supplement: Supplementary file 2 [file Table_2.DOCX]

**Supplemental Table 2.** Sequences of oligonucleotide primers used in RT-PCR, quantitative PCR, and expression of recombinant polypeptides.

| Primer Name | Sequence (5’→3’) |
| --- | --- |
| **RT-PCR for *istr1***  Istr1-SQ-F1  Istr1-SQ-R1  **RT-PCR for *istr2***  Istr2-SQ-F1  Istr2-SQ-R1  **Quantitative PCR for *gh***  GH-QF1  GH-QR1  **Quantitative PCR for *prl***  PRL-QF  PRL-QR  **Quantitative PCR for *actb***  actb-QF1  actb-QR1  **Quantitative PCR for *gapdh***  gapdh-QF  gapdh-QR  **Quantitative PCR for *hprt1***  hprt1-QF  hprt1-QR  **Expression of Istr1 antigen**  Istr1-F  Istr1-R  **Expression of Istr2 antigen**  Istr2-F  Istr2-R  **Expression of Prl antigen**  Prl-F  Prl-R  **Expression of Istr1**  pcDNA3.0-Istr1-F  pcDNA3.0-Istr1-R  **Expression of Istr2**  pcDNA3.0-Istr2-F  pcDNA3.0-Istr2-R | ATTCCTGGCTTTCACTCCCATGGTC  GGAAGAGTCTGTCAGGTACCGTCTA  AAAACCCGGCGGGAGCAGTGTATAA  GGATGACTTGAGGTAGCGCGTGGA  AAGTCATCCTCCTGCTATCAGTCCT  CTGTCTGCAGAGAGCTCTCAAAGTC  ACACTCACTCAAGAGCTGGACTC  GGGGGTCCACCCAGGATTTG  GCAGAGCCTAGACGACCAACTC  GGGTGCGTTTCTTAAACCTAGC  TCACTGCTACCCAGAAGACCG  CTCAGGAATGACCTTGCCCAC  TTGGACAGGACAGAGCGACT  TCATTGGGATGGAGCGGT  CCATGGGCATGCAGTGCTTCTGCTGCTGTA  GGATCCTTAGTGTCCTGGGCACCCTGCG  CATATGAGGCACAACTTTCTGTGCTGCT  GGATCCTTATGTAGTAGATGTCTGTGTG  GAGAGACCATGGGTCCCCATCACCGAGCTGCTTG  GAGAGACTCGAGTTAGCACATCTCAGGTTGCAGC  GACCGGAATTCGCCACCATGGAGGACATTTTACGCGAGC  GGCCGCTCGAGTTAGTGTCCTGGGCA CCCTGCG  CGCGGGGTACCACAATGGAAAGCATT TCAAATG  GGCCGCTCGAGCTATGTAGTAGATGTCTGTGTG |

F: sense primer; R: antisense primer.
