# Supplementary material for: Isotocin Regulates Growth Hormone but Not Prolactin Release From the Pituitary of Ricefield Eels
Source: Front Endocrinol (Lausanne). 2018 Apr 12;9:166. doi: 10.3389/fendo.2018.00166 (PMC5906535; doi:10.3389/fendo.2018.00166)
Supplement: Supplementary file 3 [file Data_Sheet_1.DOCX]

**SUPPLEMENTAL DATA**

**Generation of Polyclonal Antiserum against Ricefield Eel Prl**

The cDNA sequences encoding a segment of ricefield eel Prl (amino acid residues 25-212, Prl antigen) was PCR amplified using gene-specific primer set Prl-F/Prl-R. The PCR products were cloned into pET-15b via *Nco* I and *Xho* I sites and expressed in the host *E.coli* BL21(*DE3*) as recombinant protein by IPTG induction. The sequences of all primers used are listed in Supplemental Table 2. The recombinant Prl antigen was gel purified from the inclusion bodies and used to immunize rabbit and BALB/C mice as previously reported (Wu et al., 2012).

The specificity of Prl immunoreactivity in the pituitary of ricefield eel was determined by immunohistochemical analysis (Supplemental Fig. 5). The experimental methods of immunohistochemistry and fluorescent immunohistochemistry were conducted as described in Materials and Methods.

**The Measurement of Intracellular cAMP and Calcium Concentrations in Primary Pituitary Cells of Ricefield Eels**

To assay the effects of isotocin on cAMP production in primary pituitary cells of female ricefield eels, the dispersed pituitary cells were seeded in 24-well plates (Nunc, Denmark) at approximately 1×10^6^ cells/mL per well with DMEM (Gibco, MA, USA) containing 10% FBS (Gibco, MA, USA) and cultured at 28 °C with 5% CO_2_. After pre-incubation for 12 hrs, the cells were treated with isotocin (100 nM) for 12 hrs. Each treatment was repeated in four wells. After treatment, the pituitary cells per well were collected by 100 μl RIPA lysis buffer (Beyotime), and homogenized in 10 volumes of 0.1 M HCl. After centrifugation at 1000 x g for 5 min at room temperature, the supernatants were collected and the amount of cAMP in pituitary cells was quantified with a Monoclonal Anti-cAMP Antibody Based Direct cAMP ELISA Kit (catalog number 80203, NewEast Biosciences, Inc., PA, USA) by following manufacturer’s instruction. Results are expressed as measured cAMP concentrations in pituitary cell homogenates. The experiments were repeated twice, and similar results were obtained.

For measuring intracellular calcium concentrations, the dispersed pituitary cells of female ricefield eels were seeded in FluoroDish^TM^ (World Precision Instruments, Inc., USA) at approximately 1×10^6^ cells/mL per well with DMEM (Gibco, MA, USA) containing 10% FBS (Gibco, MA, USA) and cultured at 28 °C with 5% CO_2_ for 24 hrs. Then the culture medium was replaced with DMEM only. After 16-hr culture, the culture medium was removed and cells were rinsed once with HBSS (Gibco, MA, USA) and incubated in HBSS containing 5 μM fluorescent dye Fluo3-acetoxymethyl ester (Fluo-3/AM; Molecular Probes) in the dark for 45 minutes at room temperature. Subsequently, cells were rinsed three times with HBSS before intracellular Ca^2+^ measurement, and the fluorescence signal in single cells was recorded using a laser scanning confocal imaging system (TCS SP5; Leica Microsystems, Mannheim, Germany), with an excitation wavelength of 488 nm and an emission wavelength of 530 nm. Ca^2+^ levels were presented as fluorescence intensity and expressed as percentage of the initial Fluo-3 fluorescence.

**The Effects of Isotocin on Serum Gh Levels in Tilapia**

A total of 80 male tilapia (body length 10-12 cm, body weight 30-40 g) were purchased from the Guangdong National Tilapia Farm (Guangzhou, China), and kept in eight 50-litre plastic tanks in laboratory under a natural photoperiod and room temperature in November 2017, with ten fish each tank as a group. The tank water was replaced on alternate days. After acclimatization for three days, tilapia received intraperitoneal injections of either isotocin (0.1 μg/g body weight; 40 fish of four tanks) or 0.65% NaCl (vehicle control; 40 fish of the other four tanks). The serum samples of tilapia were obtained at 3, 6, 12, and 24 hrs after injection (10 fish of one tank for each treatment at each sampling time point). The Gh levels in the serum samples were analyzed with the Tilapia Growth Hormone(GH) ELISA Kit (Shanghai Enzyme-linked Biotechnology Co., Ltd., Shanghai, China).
