# Supplementary material for: Isotocin Regulates Growth Hormone but Not Prolactin Release From the Pituitary of Ricefield Eels
Source: Front Endocrinol (Lausanne). 2018 Apr 12;9:166. doi: 10.3389/fendo.2018.00166 (PMC5906535; doi:10.3389/fendo.2018.00166)
Supplement: Supplementary file 4 [file Data_Sheet_2.PDF]

Supplemental Fig. 1

```
                                                                                   TMD1
Ricefield Istr1       MEDILRE--QDNWAQNISWNNSSLGNDSDLGNTTVNPLKRNEEVAKVEVTVLVLVLLLAL 58
Tilapia Istr 1        MEDLLRE--QYSWSHNLTWSSSSRENESHVGNATVNPLKRNEEVAKVEVTVLVLVLLLAL 58
Ricefield Istr 2      MESTSNE--SDFW-FNDSWRNSSLLNGTD-GMNQTNPLKRNEEVAKVEVTVLALVLFLAL 56
Tilapia Istr 2        MESISNE--SDIWQFNESWRNSSLINGTG-GLNQTNPLKRNEEVARVEVTVLALVLFLAL 57
Xenopus Oxtr          MEPLCAQPDCPLLLLNGSWGNFSMENRTGPTNTTRDPLKRNEDVAKVEVAVLALILFLAL 60
Mouse Oxtr            MEGTPAA-NWSIELDLGSGVPPGAEG-----NLTAGPPRRNEALARVEVAVLCLILFLAL 54
Human OXTR            MEGALAA-NWSAEAANASAAPPGAEG-----NRTAGPPRRNEALARVEVAVLCLILLLAL 54
                      **               :    .  .         .* :*** :*:***:** *:*:***

                                                        TMD2
Ricefield Istr1       TGNLCVLWAINTTKHSQSRMYYFMKHLSIADLVVAIFQVLPQLIWDITFRFYGPDLLCRL 118
Tilapia Istr1         TGNLCVLWAIHTTKHSQSRMYYFMKHLSIADLVVAIFQVLPQLIWDITFRFYGPDLLCRL 118
Ricefield Istr2       AGNMCVLLAIHTTKHSQSRMYYFMKQLSIADLVVAIFQVLPQLIWDITFRFYGPDILCRL 116
Tilapia Istr2         AGNLCVLLAIHTTKHSQSRMYYFMKHLSIADLVVAIFQVLPQLIWDITFRFYGPDILCRL 117
Xenopus Oxtr          AGNICVLIAIHINRHKHSRMYFFMKHLSIADLVVALFQVLPQLIWDITFRFYAPDFVCRI 120
Mouse Oxtr            SGNACVLLALRTTRHKHSRLFFFMKHLSIADLVVAVFQVLPQLLWDITFRFYGPDLLCRL 114
Human OXTR            SGNACVLLALRTTRQKHSRLFFFMKHLSIADLVVAVFQVLPQLLWDITFRFYGPDLLCRL 114
                      :** *** *:. .::.:**:::***:*********:*******:********.**::**:

                          TMD3                                  TMD4
Ricefield Istr1       VKYLQVVGMFASTYMLVLMSVDRCLAIWQPLRSLHKRKDRFCVITSWLLSLIFSTPQLYI 178
Tilapia Istr1         VKYLQVVGMFASTYMLVLMSIDRCLAVCQPLRSVHRKKDRFCVIASWMLSLIFSSPQAYI 178
Ricefield Istr2       VKYLQVVGMFASTYMLVLMSVDRCLAICQPLRSLHRRKDRFYVIFSWVLSLLFSIPQMFI 176
Tilapia Istr2         VKYLQVVGMFASTYMLVLMSIDRCLAICQPLRSLHRRKDRLYVIFSWILSLLFSIPQMFI 177
Xenopus Oxtr          VKYLQVVGMFASTYMLLLMSLDRCLAICQPLRSLHRRSDCVYVVLTWILSFLLSVPQIHI 180
Mouse Oxtr            VKYLQVVGMFASTYLLLLMSLDRCLAICQPLRSLRRRTDRLAVLATWLGCLVASVPQVHI 174
Human OXTR            VKYLQVVGMFASTYLLLLMSLDRCLAICQPLRSLRRRTDRLAVLATWLGCLVASAPQVHI 174
                      **************:*:***:*****: *****::::.* . *: :*: .:: * ** .*

                                                             TMD5
Ricefield Istr1       FSLKEVG---NGVYDCWGDFVQPWGAKAYITWMTLTIYLLPVAILSTCYGLICFKIWQNF 235
Tilapia Istr1         FSLREVG---NGVYDCWGDFVQPWGAKAYITWMSLSIYIFPVAILSICYGLICFKIWENF 235
Ricefield Istr2       FSLREVGSAGSGVYDCWGDFVKPWGAKAYITWISLTIYIIPVAILSICYGLISFKIWQNF 236
Tilapia Istr2         FSLREVGSAGSGVYDCWGDFVKPWGAKAYITWISLTIYIIPVAILSICYGLISFKIWQNF 237
Xenopus Oxtr          FSLKEVG---NKVYDCSASFIEPWGLKTYITWITITVYILPVMILSVCYGLISYKIWQNI 237
Mouse Oxtr            FSLREVA---DGVFDCWAVFIQPWGPKAYVTWITLAVYIVPVIVLAACYGLISFKIWQNL 231
Human OXTR            FSLREVA---DGVFDCWAVFIQPWGPKAYITWITLAVYIVPVIVLAACYGLISFKIWQNL 231
                      ***:**.   . *:** . *::*** *:*:**:::::*:.** :*: *****.:***:*:

                                                                  TMD6
Ricefield Istr1       DLKTRRKPFLAFTPMVAKG---AHPLSRVSSVGLISKAKMRTVKMTFVVVLAYTVCWTPF 292
Tilapia Istr1         NLKTRREHFLALTPRPSKG---AQPLSRVSSVRLISKAKIRTVKMTFVVVLAYIVCWTPF 292
Ricefield Istr2       KLKTRREQCISLTPKTSK----GNTLARVSSVKLISKAKITTVKMTFVIVVAYIVCWTPF 292
Tilapia Istr2         KLKTRREQCINLTPKTTK----SNTLARVSSVKLISKAKITTVKMTFVIVVAYIVCWTPF 293
Xenopus Oxtr          RLKTMCESSVRLSS------NKRATLSRVSSVRLISKAKIRTVKMTFIIVLAYIVCWTPF 291
```

```
Mouse Oxtr           RLKTAAAAAAAEGSDAAGG-AGRAALARVSSVKLISKAKIRTVKMTFIIVLAFIVCWTPF 290
Human OXTR           RLKTAAAAAAEAPEGAAAGDGGRVALARVSSVKLISKAKIRTVKMTFIIVLAFIVCWTPF 291
                      ***                    .*:***** ******: ******::*:*: ******

                                                TMD7
Ricefield Istr1      FFVQMWSAWDPAAPREDTAFIIAMLLASLNSCCNPWIYLSFAGHMFHDLMQC-FCCCRRY 351
Tilapia Istr1        FFVQMWSAWDPAAPREDMAFIIAMLLASLNSCCNPWIYMFFAGHLFHDLMQCFFCCCRRY 352
Ricefield Istr2      FSVQMWSAWDPEAPREAMPFIISMLLASLNSCCNPWIYMCFAGHLFQDLRHNFLCCSTRY 352
Tilapia Istr2        FSVQMWSAWDPAAPREAMPFIISMLLASLNSCCNPWIYMCFAGHLFQDLRQNLLCCSTRY 353
Xenopus Oxtr         FFVQMWSVWDPDAPKEDSLFIIAMLLGSLNSCCNPWIYMLFTGHLFHDLLQRFLCCSARY 351
Mouse Oxtr           FFVQMWSVWDVNAPKEASAFIIAMLLASLNSCCNPWIYMLFTGHLFHELVQRFLCCSARY 350
Human OXTR           FFVQMWSVWDANAPKEASAFIIVMLLASLNSCCNPWIYMLFTGHLFHELVQRFLCCSASY 351
                     * *****.**  **:*   *** ***.***********: *:**:*::* :  :**.  *

Ricefield Istr1      LTDSSCSCDQRCK--QKSNPSTCVIKN-TSSHRSLTYICSAGCPGH 394
Tilapia Istr1        LTECSCSCDQQCR--HKRSSSTYVNKN-TNSQRSLSRTSSTVH--- 392
Ricefield Istr2      LKSSQCKCERDFDSSHKSNSSTFAMKS-TSSQRSVTQTSTT----- 392
Tilapia Istr2        LKSSQCHCERDFNSSHKSNSSTFAIKS-TSSQRSITQTSTT----- 393
Xenopus Oxtr         LKSSQQGSDMSTS--RKSNSSTFVLSRKSSSQKSITQPSIA----- 390
Mouse Oxtr           LKGSRPG-ETSIS--KKSNSSTFVLSRRSSSQRSCSQPSSA----- 388
Human OXTR           LKGRRLG-ETSAS--KKSNSSSFVLSHRSSSQRSCSQPSTA----- 389
                     *.       :      :* ..*: . .  :.*::* :  . :
```

Supplemental Figure 1. Alignment (by ClustalX 1.83) of the amino acid sequences of ricefield eel isotocin receptors with those of Istrs or Oxtrs of other vertebrates. The identical, highly conserved, and less conserved amino acid residues were indicated by (*), (:), and (.), respectively. The amino acid sequences highlighted by the gray background are the transmembrane regions. The protein sequences of other vertebrate Istrs or Oxtrs were downloaded from *Entrez* (NCBI) with following accession numbers: Tilapia Istr1 (XP_003448447), Tilapia Istr2 (XP_003441492), Xenopus Oxtr (XP_002936297), Mouse Oxtr (NP_001074616.1), and Human OXTR (AAI37444.1).
