# Supplementary material for: Isotocin Regulates Growth Hormone but Not Prolactin Release From the Pituitary of Ricefield Eels
Source: Front Endocrinol (Lausanne). 2018 Apr 12;9:166. doi: 10.3389/fendo.2018.00166 (PMC5906535; doi:10.3389/fendo.2018.00166)
Supplement: Supplementary file 5 [file Data_Sheet_3.PDF]

Supplemental Fig. 2

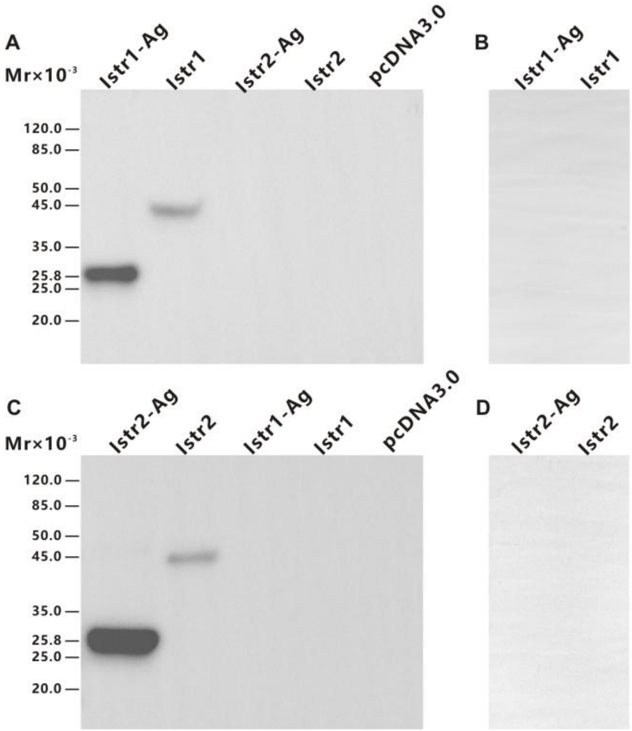

Supplemental Figure 2. Specificities of anti-Istr1 and anti-Istr2 antisera examined by Western blot analysis. The recombinant Istr antigens (200 ng) and full-length proteins (in COS-7 cell extracts, 500 μg) were separated on 12 % SDS-PAGE gels, transferred to polyvinylidene fluoride membranes, and immnoreacted with the rabbit anti-Istr1 antiserum (1: 1000; A), the pre-absorbed rabbit anti-Istr1 antiserum by recombinant Istr1 antigen (1:1000; B), the rabbit anti-Istr2 antiserum (1:1000; C) or the pre-absorbed rabbit anti-Istr2 antiserum by recombinant Istr2 antigen (1:1000; D). Istr1 antigen, the recombinant Istr1 polypeptide used to immunize rabbits; Istr2 antigen, the recombinant Istr2 polypeptide used to immunize rabbits. Istr1 and Istr2, the recombinant full-length Istr1 and Istr2 proteins expressed in transiently transfected COS-7 cells. The secondary antibodies were 1:5000 diluted horseradish peroxidase (HRP)-conjugated goat anti-rabbit IgG (H+L) (catalog number: 111-035-003, Jackson ImmunoResearch Laboratories, Inc., PE, USA). The blots were visualized using the BeyoECL Plus kit (Beyotime).
