# Supplementary material for: Isotocin Regulates Growth Hormone but Not Prolactin Release From the Pituitary of Ricefield Eels
Source: Front Endocrinol (Lausanne). 2018 Apr 12;9:166. doi: 10.3389/fendo.2018.00166 (PMC5906535; doi:10.3389/fendo.2018.00166)
Supplement: Supplementary file 6 [file Data_Sheet_4.PDF]

Supplemental Fig. 3

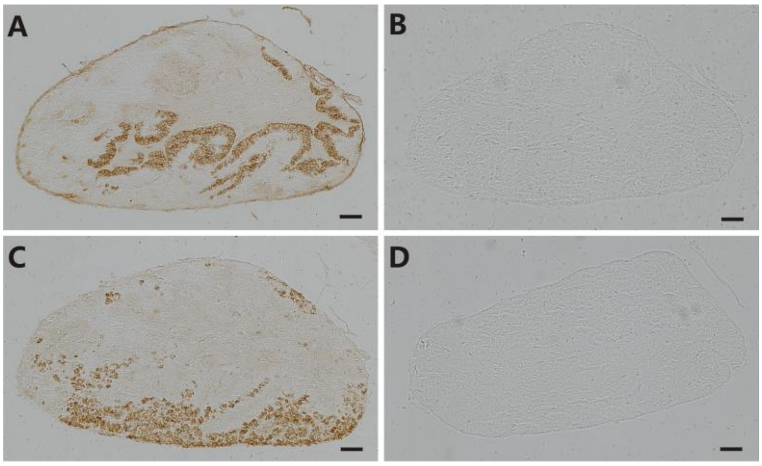

Supplemental Figure 3. Cellular localization of immunoreactive Istr1 and Istr2 in the pituitary of female ricefield eels. The rabbit anti-Istr1 antiserum (1:500, A), the pre-absorbed rabbit anti-Istr1 antiserum by the excessive Istr1 recombinant protein (1:500, B), the rabbit anti-Istr2 antiserum (1:500, C), and the pre-absorbed rabbit anti-Istr2 antiserum by the excessive Istr2 recombinant protein (1: 500, D) were used as primary antisera, respectively. The secondary antibody was 1:500 diluted horseradish peroxidase (HRP)-conjugated goat anti-rabbit IgG (H+L) (catalog number: 111-035-003; Jackson ImmunoResearch Laboratories, Inc., PE, USA). The immunohistochemistry sections were visualized by DAB chromogen. Scale bar is 50 μm.
