# Supplementary material for: Isotocin Regulates Growth Hormone but Not Prolactin Release From the Pituitary of Ricefield Eels
Source: Front Endocrinol (Lausanne). 2018 Apr 12;9:166. doi: 10.3389/fendo.2018.00166 (PMC5906535; doi:10.3389/fendo.2018.00166)
Supplement: Supplementary file 7 [file Data_Sheet_5.PDF]

Supplemental Fig. 4

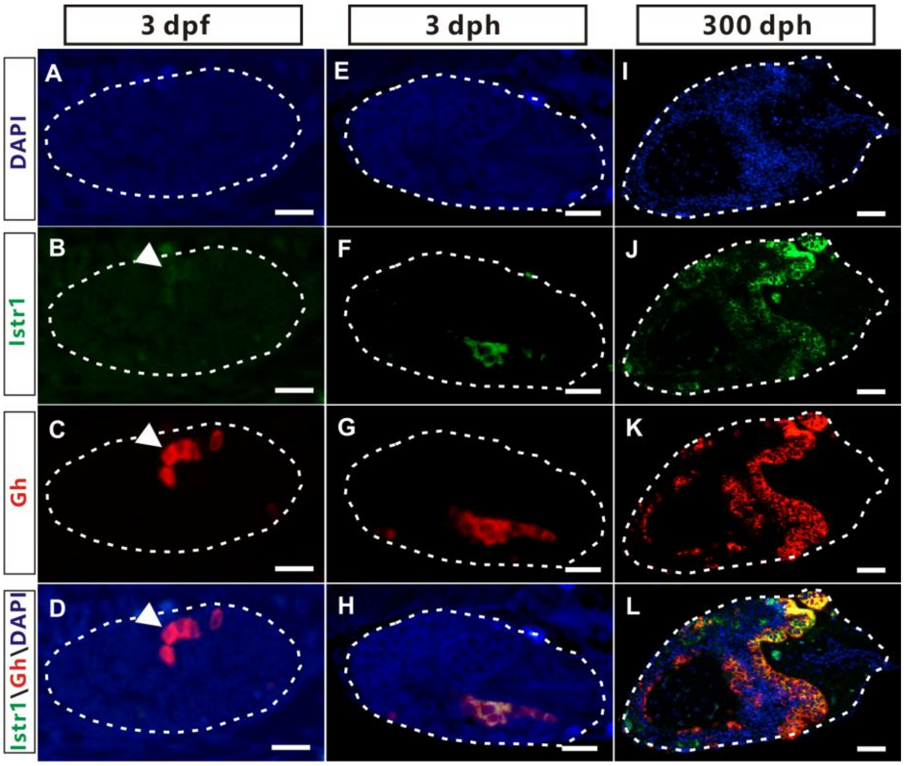

Supplemental Figure 4. The co-localization of immunoreactive Istr1 (green) and Gh (red) in the pituitary of ricefield eels at early developmental stages of 3 days post fertilization (dpf), 3 days post hatching (dph), and 300 dph. The rabbit antiserum against Istr1 (1:500) and mouse antiserum against Gh (1:800) were used as primary antisera. The secondary antibodies were 1:500 diluted Alexa Fluor 488-labeled goat anti-rabbit IgG (H+L) for Istr1, and 1:500 diluted Cy3-labeled goat anti-mouse lgG (H+L) for Gh. DAPI was used to stain the nuclei blue. Sagittal sections of ricefield eel pituitary glands were shown here with the rostral (anterior) to the left. The images were observed and captured with a confocal microscope under the same conditions. The pituitary glands are delineated with dashed lines. The white triangles indicate the position of positive signals at 3 dpf. Scale bar is 50 μm.
