# Supplementary material for: Isotocin Regulates Growth Hormone but Not Prolactin Release From the Pituitary of Ricefield Eels
Source: Front Endocrinol (Lausanne). 2018 Apr 12;9:166. doi: 10.3389/fendo.2018.00166 (PMC5906535; doi:10.3389/fendo.2018.00166)
Supplement: Supplementary file 8 [file Data_Sheet_6.PDF]

Supplemental Fig. 5

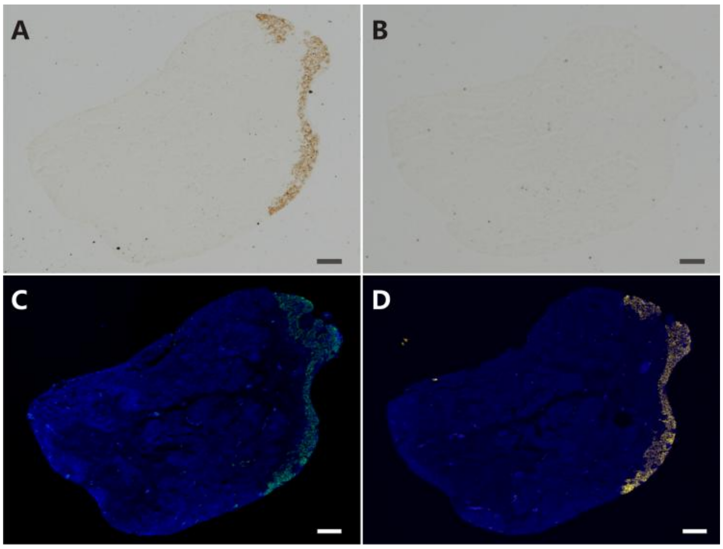

Supplemental Figure 5. The specificity of Prl immunoreactivity in the pituitary of ricefield eels as determined by immunohistochemical analysis. A, immunostaining with the rabbit anti-Prl antiserum as primary antibody and visualized by DAB chromogen. B, immunostaining with the rabbit anti-Prl antiserum (1:500) pre-absorbed by excessive Prl antigen and visualized by DAB chromogen. C, immunostaining with rabbit anti-Prl antiserum (1:500) as primary antibody and visualized by Alexa Fluor 488 fluorescence. D, immunostaining with rabbit anti-Prl antiserum (1:500) and mouse anti-Prl antiserum (1:800) as primary antibodies and visualized by Alexa Fluor 488 and Cy3 fluorescence, respectively. The nuclei were stained blue with DAPI. Sagittal sections of the pituitary were shown here with the rostral (anterior) to the left. Scale bar is 50 μm.
