# Supplementary material for: Isotocin Regulates Growth Hormone but Not Prolactin Release From the Pituitary of Ricefield Eels
Source: Front Endocrinol (Lausanne). 2018 Apr 12;9:166. doi: 10.3389/fendo.2018.00166 (PMC5906535; doi:10.3389/fendo.2018.00166)
Supplement: Supplementary file 9 [file Data_Sheet_7.PDF]

Supplemental Fig. 6

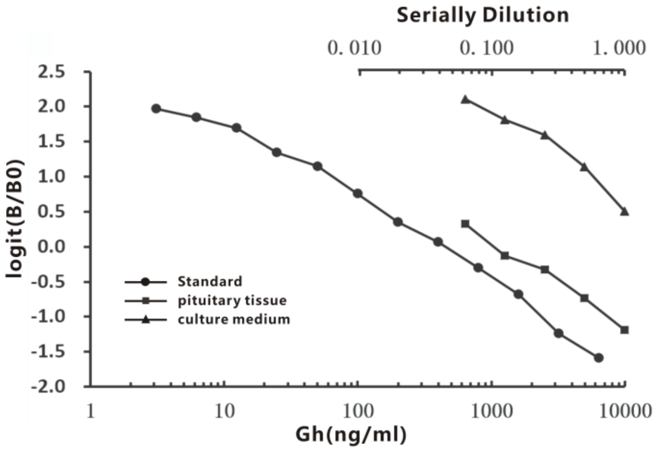

Supplemental Figure 6. Correlation between the standard curve of the competitive ricefield eel Gh ELISA and the dilution curves of the pituitary homogenate and culture medium. Round, square, and triangle represent the dilution curves of the standard, pituitary homogenate and culture medium, respectively. The abscissa is the logarithm of the concentration (ng/mL), and the ordinate is the logarithm of the binding rate (B/B0). The detection limit of the assay was 3.1 ng/ml (R^2=0.997), and the ED_{50} value was 396.0 ng/ml. The intraassay CV and interassay CV of the assay system were less than 6.6% and 8.4%, respectively, which are within acceptable parameters.
