# Supplementary material for: Isotocin Regulates Growth Hormone but Not Prolactin Release From the Pituitary of Ricefield Eels
Source: Front Endocrinol (Lausanne). 2018 Apr 12;9:166. doi: 10.3389/fendo.2018.00166 (PMC5906535; doi:10.3389/fendo.2018.00166)
Supplement: Supplementary file 10 [file Data_Sheet_8.PDF]

Supplemental Fig. 7

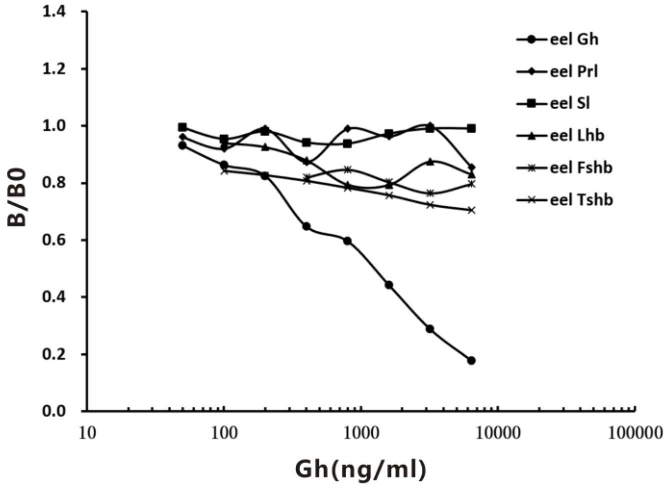

Supplemental Figure 7. Cross reactivities of ricefield eel Gh ELISA with recombinant ricefield eel Prl, Sl, Lhb, Fshb, and Tshb. The lines of different symbols represent the dilution curves of Gh standard, Prl, Sl, Lhb, Fshb, and Tshb recombinant proteins, respectively. The abscissa is the logarithm of Gh concentrations (ng/mL), and the ordinate is the binding rate (B/B0). Ricefield eel Gh ELISA showed less than 0.01% cross-reactivities with the other pituitary hormones tested.
