# Supplementary material for: Isotocin Regulates Growth Hormone but Not Prolactin Release From the Pituitary of Ricefield Eels
Source: Front Endocrinol (Lausanne). 2018 Apr 12;9:166. doi: 10.3389/fendo.2018.00166 (PMC5906535; doi:10.3389/fendo.2018.00166)
Supplement: Supplementary file 11 [file Data_Sheet_9.PDF]

Supplemental Fig. 8

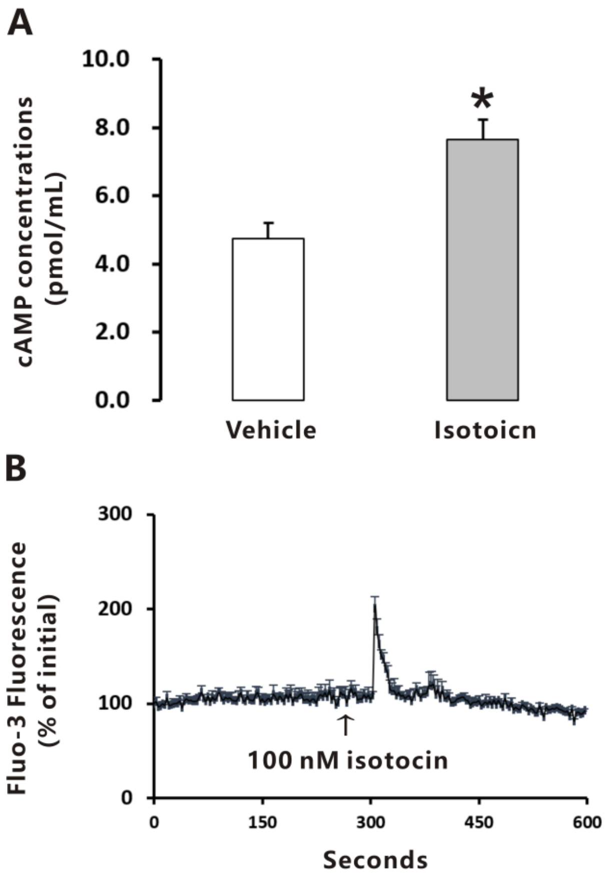

Supplemental Figure 8. Activation of cAMP and Ca^{2+} signaling pathways by isotocin in primary pituitary cells of female ricefield eels. A, effects of isotocin on cellular cAMP contents. The pituitary cells were pre-incubated for 12 hrs before being treated with isotocin (100 nM) for 12 hrs. After treatment, the amount of cAMP in the pituitary cells was quantified with a Monoclonal Anti-cAMP Antibody Based Direct cAMP ELISA Kit (catalog number 80203, NewEast Biosciences, Inc., PA, USA). Results are expressed as measured cAMP concentrations in pituitary cell homogenates. Bars represent means ± SEM (n=4). *P<0.05 vs the vehicle control. B, effects of isotocin on intracellular Ca^{2+} levels. The primary pituitary cells were plated onto cell culture dish, loaded with 5 μM Fluo-3 AM. The fluorescence intensity in single cells was analyzed with a laser scanning confocal imaging system (TCS SP5; Leica Microsystems, Mannheim, Germany), and expressed as percentage of the initial Fluo-3 fluorescence. Ca^{2+} levels are presented as mean fluorescence intensities ± SEM (n=3) of three independent experiments.
