# Supplementary material for: Isotocin Regulates Growth Hormone but Not Prolactin Release From the Pituitary of Ricefield Eels
Source: Front Endocrinol (Lausanne). 2018 Apr 12;9:166. doi: 10.3389/fendo.2018.00166 (PMC5906535; doi:10.3389/fendo.2018.00166)
Supplement: Supplementary file 12 [file Data_Sheet_10.PDF]

Supplemental Fig. 9

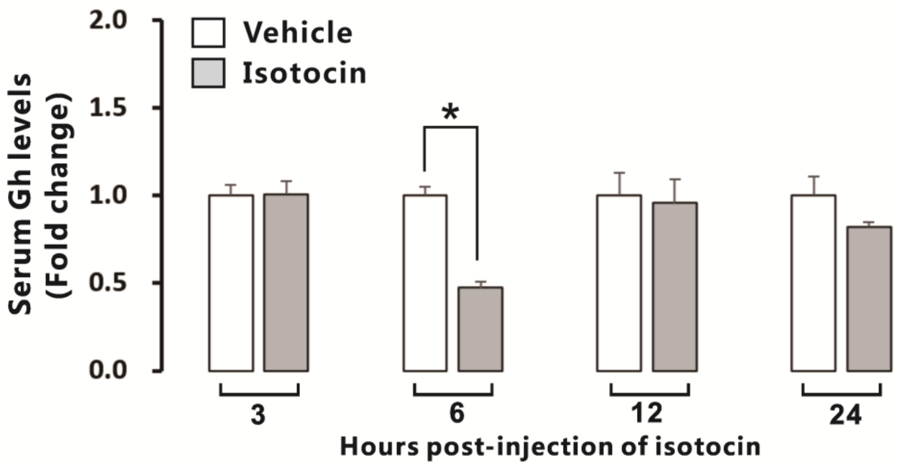

Supplemental Figure 9. The relative growth hormone (Gh) levels in the serum of tilapia at 3, 6, 12, and 24 hrs after intraperitoneal injection of isotocin (0.1 μg/g body weight) or 0.65% NaCl (vehicle control). After injection, the level of Gh in the serum was quantified with a Tilapia Growth Hormone (GH) ELISA Kit (catalog number SU-B90103, Shanghai Enzyme-linked Biotechnology Co., Ltd., Shanghai, China). Data were expressed as fold change relative to the corresponding vehicle control. Bars represent means ± SEM (n=10). *P ＜0.05 vs. the corresponding control.
